# Comparative evaluation of CAM methods for enhancing explainability in veterinary radiography

**DOI:** 10.1038/s41598-025-14060-6

**Published:** 2025-08-13

**Authors:** Piotr Dusza, Tommaso Banzato, Silvia Burti, Margherita Bendazzoli, Henning Müller, Marek Wodzinski

**Affiliations:** 1 AGH University of Krakow, Faculty of Electrical Engineering, Automatics, Computer Science and Biomedical Engineering, Krakow, 30059 Poland; 2 University of Applied Sciences Western Switzerland (HES-SO), Institute of Informatics, Sierre, 3960 Switzerland; 3 University of Padua, Department of Animal Medicine, Production and Health, Legnaro, 35020 Padua Italy; 4 University of Geneva, Medical Faculty, Geneva, Switzerland; 5 The Sense Research and Innovation Center, Sion, Lausanne Switzerland

**Keywords:** Interpretability, Explainability, GradCAM, XAI, Deep learning, Veterinary, Health care, Oncology

## Abstract

Explainable Artificial Intelligence (XAI) encompasses a broad spectrum of methods that aim to enhance the transparency of deep learning models, with Class Activation Mapping (CAM) methods widely used for visual interpretability. However, systematic evaluations of these methods in veterinary radiography remain scarce. This study presents a comparative analysis of eleven CAM methods, including GradCAM, XGradCAM, ScoreCAM, and EigenCAM, on a dataset of 7362 canine and feline X-ray images. A ResNet18 model was chosen based on the specificity of the dataset and preliminary results where it outperformed other models. Quantitative and qualitative evaluations were performed to determine how well each CAM method produced interpretable heatmaps relevant to clinical decision-making. Among the techniques evaluated, EigenGradCAM achieved the highest mean score and standard deviation (SD) of 2.571 (SD = 1.256), closely followed by EigenCAM at 2.519 (SD = 1.228) and GradCAM++ at 2.512 (SD = 1.277), with methods such as FullGrad and XGradCAM achieving worst scores of 2.000 (SD = 1.300) and 1.858 (SD = 1.198) respectively. Despite variations in saliency visualization, no single method universally improved veterinarians’ diagnostic confidence. While certain CAM methods provide better visual cues for some pathologies, they generally offered limited explainability and didn’t substantially improve veterinarians’ diagnostic confidence.

## Introduction

The rapid advancement of artificial intelligence (AI), especially deep learning (DL), has led to considerable advances in various sectors, including healthcare. Within this domain, convolutional neural networks (CNNs) are increasingly integrated into medical imaging applications^1^, offering substantial improvements in diagnostic accuracy and efficiency. These advances address long-standing bottlenecks in medical diagnostics, such as the precision and speed of diagnosis^2^ that have historically been limited by information flow restrictions arising from communication problems^3–6^. However, a critical challenge persists in the form of limited interpretability and explainability of DL models, as they do not readily reveal how they arrive at their prediction. Often described as “black boxes”^7^, these systems lack transparency, making it difficult for clinicians to fully trust and communicate the reasoning behind diagnostic decisions. To address this, explainable artificial intelligence (XAI) methods aim to make AI outputs more interpretable, enabling clinicians to better understand and trust the models’ predictions.

However, while human medicine embraced the changes and advances were slowly made, veterinary medicine lagged behind, with researchers emphasizing that the extent of AI in veterinary medicine should be increased^8–11^. Although the push for such advances is promising, as CNNs have shown impressive accuracy in image analysis, the explainability of these models remains a considerable challenge. Problems with the explainability of CNNs arise from their closed nature that is usually referred to in the literature as a “black box”^7^. Often, as a result, its clinical adoption is hampered, as the ability to understand the ’decision-making’ and ’reasoning’ processes of the model are crucial for clinical trust and diagnostic processes^7,12,13^. This necessity extends beyond veterinary medicine and is critical in all branches of medicine. Regardless of their specialization or patient, clinicians must understand why AI systems make specific predictions to build trust in their reliability and decision-making processes.

Explainable artificial intelligence (XAI) seeks to mitigate this issue by providing tools that elucidate the output of the AI model^14^. Among these tools, saliency methods, such as Class Activation Mapping (CAM), have become instrumental in medical diagnostics. CAM methods are utilized to generate heatmaps that highlight influential regions within medical images, which then helps clinicians determine whether a correct area influenced the classifier’s decision^12,14^. By highlighting only the relevant structures, these visual explanations can potentially boost physicians’ trust in the predictions made by deep learning models.

The primary advantage of CAMs lies in their ability to provide visual interpretations that are relatively easy for clinicians to understand. By overlaying heatmaps on medical images, CAMs highlight areas of interest, facilitating a more intuitive understanding for clinicians. This capability allows for a clearer interpretation of model predictions, as it visually delineates the areas that contribute the most to the classification decision, making the decision-making process more transparent^15–17^. Additionally, CAM’s integration with existing CNN architectures is relatively seamless as it requires no modifications to the network. This simplicity allows for rapid deployment without the need for retraining^18^. Furthermore, CAM’s ability to produce class-specific heatmaps enhances its utility in scenarios where distinguishing between multiple pathology classes is essential. This feature allows clinicians not only to see which areas of an image are important for a specific diagnosis but also to compare the relevance of different regions across various pathologies^19,20^.

However, CAMs also have notable disadvantages. The main limitation is related to the resolution of the intermediate feature space in CNNs, where the spatial resolution decreases after each strided convolution or pooling layer. As a result, when these heatmaps are upscaled to match the input image size, they often indicate a rough area of the pathology, without specifically pinpointing it. This limitation reduces their utility in cases that require accurate localization, as is the case with minor and confined pathologies^21^. Another significant drawback is the proliferation of various CAM methods, such as GradCAM and others that can create confusion about which method is most appropriate for a given task, further complicating their clinical adoption. Furthermore, CAM-generated heatmaps lack semantic understanding, limiting their utility. They highlight important regions, but do not offer high-level semantic explanations, which poses challenges in clinical settings where clinicians need to understand the location, nature, and implications of the pathology^21^.

Given the previously mentioned abundance of CAM methods, each with unique algorithms and outputs, there is a pressing need for peer review and comparative studies to determine which methods are most desirable to physicians. With numerous variants available, clinicians and researchers face confusion as to which method offers the best performance in a clinical setting. Speculation and deficient previous research have even debunked the perceived superiority of certain CAM methods over others^14^.

Furthermore, previous research has predominantly focused on human or even non-medical imaging, with little exploration of the application of CAMs in veterinary imaging^22^. Moreover, there has been a lack of comprehensive comparative analysis of CAM methods using clinical images^22^. Limited previous research on the subject has shown that while GradCAM (one of the CAMs) generally outperforms some commonly used saliency methods in localizing pathologies, it still falls short compared to human experts, particularly in cases involving smaller or more complex-shaped pathologies^13^. However, the research is based on human medicine, and the veterinary domain remains vastly unexplored when considering explainability and, more specifically, CAMs, highlighting the need for targeted research.

Despite these challenges, CAMs have the potential to increase the confidence of physicians in deep learning (DL) predictions. Enhancing the transparency of the AI model’s decision-making process, they can better understand and verify the results^7^. Visual explanations serve as a bridge between complex AI models and clinical reasoning, encouraging a collaborative approach where AI supports rather than replaces human knowledge^9^.

Therefore, in this article, we aim to address these gaps by investigating whether different CAMs significantly impact the explainability of CNN outputs in veterinary radiography, a domain where such comparative studies are notably sparse. Specifically, our goal is to determine to what extent veterinarians perceive differences in CAM methods in terms of color distribution and localization accuracy. By examining the application of various CAM methods on a dataset of canine X-ray images, we identify potential trends and clinicians’ preferences related to the most effective CAM method - if it exists.

*Contribution* In this article, we present a comprehensive comparative analysis of multiple CAM methods applied to a 2D canine X-ray image veterinary dataset. The evaluation of the effectiveness of each method in improving the explainability of CNN outputs is based on the color distribution and localization accuracy of the generated heatmap, from the perspective of veterinarians. Furthermore, our research evaluates how different CAM methods affect veterinarians’ confidence in AI-assisted diagnoses. In pursuit of these objectives, we contribute to the implementation of XAI in veterinary medicine, promoting greater confidence and adoption of AI technology among veterinarians.

Therefore, the article attempts to answer the following questions: Are there statistically significant differences in mean scores among various CAM methods in veterinary imaging? Does the behavior depend on a specific class? Are the CAM methods useful for veterinary doctors?

## Results

### Model performance

**Fig. 1:**
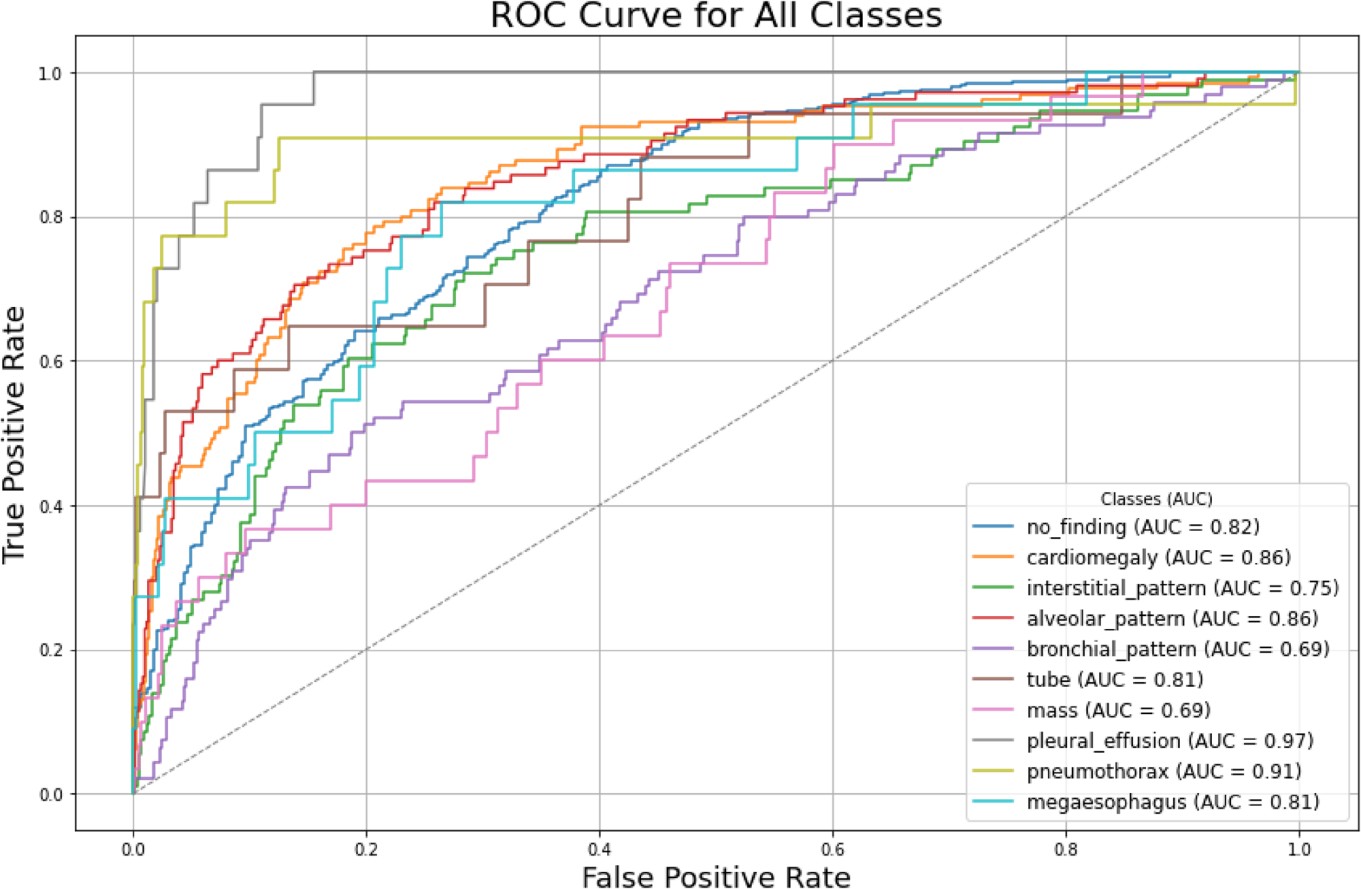
Receiver operating characteristic (ROC) curves and their corresponding area under the curve (AUC) scores for the trained ResNet18 model are presented for each of the 10 classes, which include one base class (healthy subject) that does not indicate a pathology.

Before evaluating the various CAM methods, it was essential to evaluate how well the CNN model performed in classifying different pathologies in canine X-ray images. The CNN model used in this study used the ResNet-18 architecture, selected for its superior performance in preliminary evaluations over the ResNet50 and EfficientNetV2. The difference in the preliminary performances of the models was dictated mainly by the size of the dataset (Section "Dataset"), which favored a smaller model. The ResNet-18 architecture training phase was performed using canine lateral (LL) X-ray images that were resized to 224$\times$224 pixels and normalized prior to input. Data augmentation techniques were applied probabilistically to enhance the model’s robustness while preserving critical diagnostic features. The model demonstrated satisfactory classification performance across the ten pathology classes (listed in Table 4), as evidenced by the receiver operating characteristics (ROC) curves Fig. 1.

### Relative scores

To comprehensively understand the performance of different CAM methods, it was essential to quantify their interpretability across different pathology classes. Expert veterinarian annotators evaluated the heatmaps generated by each method by first selecting the heatmap that performed best for a given set. They then assigned relative scores to the remaining methods based on two key criteria: color distribution and localization accuracy. These scores were divided to evaluate the usefulness of each method in highlighting diagnostically significant regions within the X-ray images, assessing both the relevance of activation intensity (color) and whether the heatmap accurately pinpointed the intended locations in the eyes of veterinarians.

Interestingly, the analysis did not reveal significant differences between the relative scores for color and localization, suggesting that veterinarians did not perceive a substantial distinction between methods in these aspects. Furthermore, a strong agreement was observed between the color and localization grading scores assigned by the annotators, with a mean agreement of 0.780 (at* p* = 1.074$\times$10−30). This finding suggests uniformity in visual outputs across CAM methods from a clinical perspective, leading to averaging color and localization scores for each method’s overall performance.

The following evaluation delves into these scores and explores their broader implications for improving explainability in veterinary diagnostics.

#### Evaluation of CAM methods

A comprehensive evaluation of eleven CAM methods was conducted to assess their effectiveness in enhancing the interpretability of CNN outputs in veterinary radiography. The evaluated methods were GradCAM^23,24^, HiResCAM^24,25^, GradCAMElementWise^24^, GradCAM++^24,26^, XGradCAM^24,27^, AblationCAM^24,28^, ScoreCAM^24,29^, EigenCAM^24,30^, EigenGradCAM^24^, LayerCAM^24,31^, FullGrad^24,32^.

**Fig. 2:**
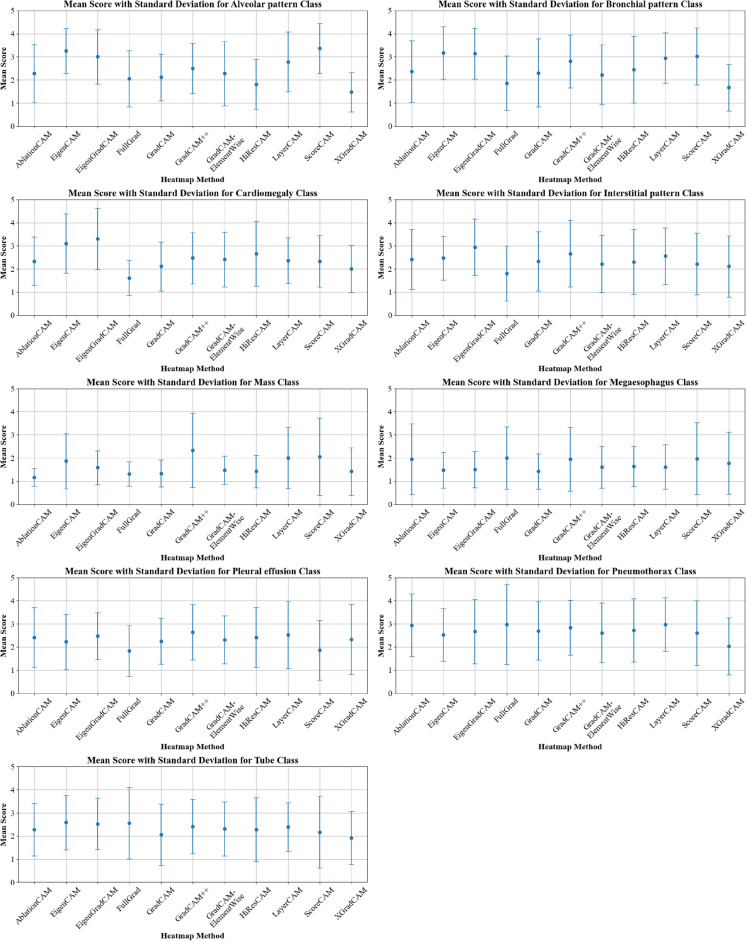
Comparison of mean scores with standard deviations for various pathology classes across multiple CAM methods.

The mean scores and standard deviations for each method, grouped by heatmap and pathology class, are shown in Fig. 2 Table. These scores represent the average evaluations of each method provided by expert annotators (practicing veterinary doctors) regarding the interpretability and relevance of the generated heatmaps.

Based on Fig. 2, it is clear that methods such as ScoreCAM, EigenCAM, and EigenGradCAM consistently achieved higher mean scores across multiple classes. For instance, in the “Alveolar pattern” class, ScoreCAM had a mean score of 3.361; however, considering the standard deviation (SD = 1.073), that score becomes less meaningful. Similarly, EigenCAM and EigenGradCAM demonstrated high mean scores in classes such as “Cardiomegaly”, “Interstitial pattern”, and “Bronchial pattern”, yet still showed high values of SD. This indicates that although a better method can be chosen per pathology, statistically, it will not be significant compared to other “worse” methods.

**Fig. 3:**
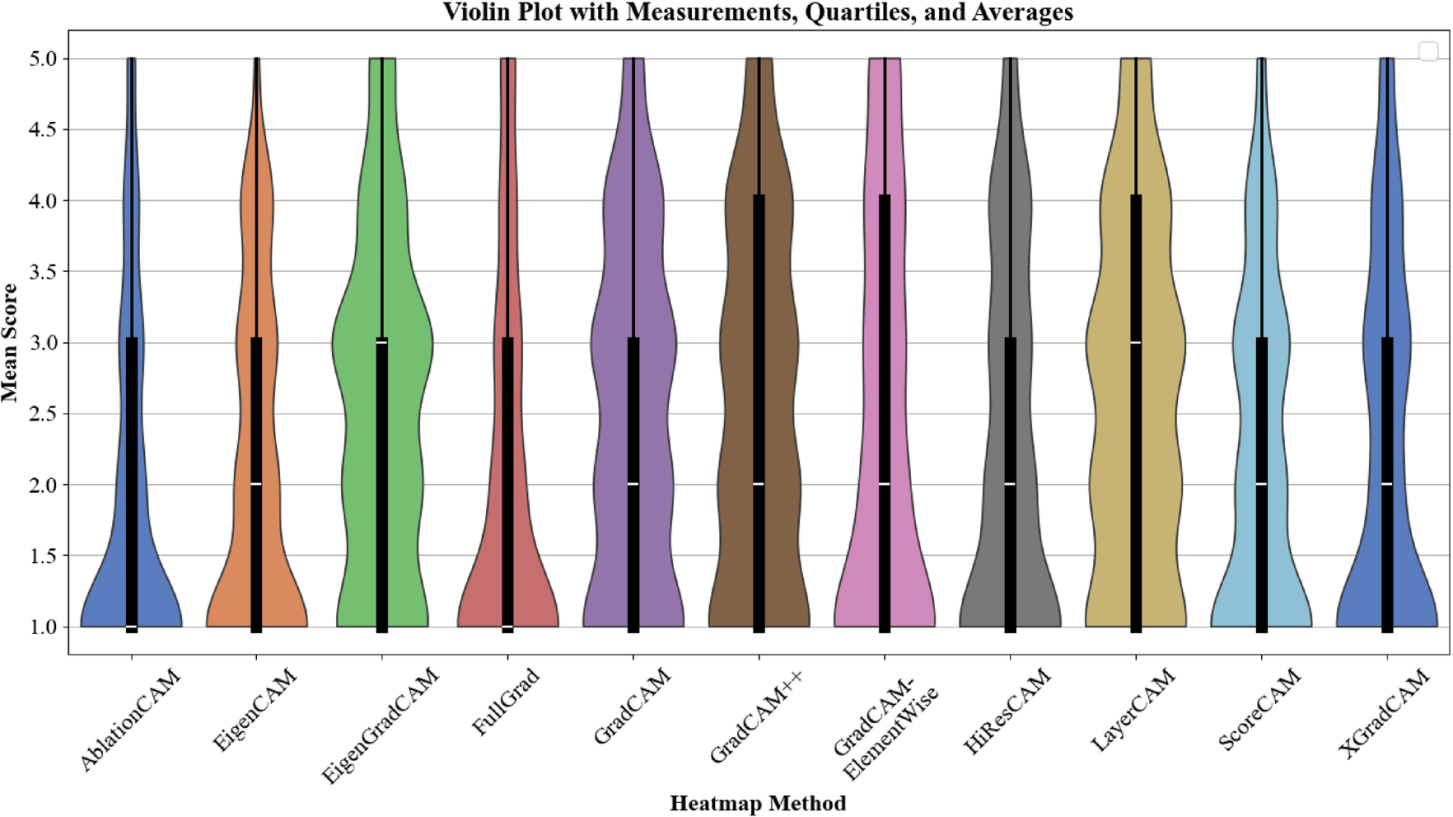
Violin plots indicating the results of various CAM methods (scores combined from all pathologies).

Furthermore, a violin plot was generated to visualize the score distributions for each CAM method across all pathology classes, as shown in Fig. 3. This plot provides a dual perspective by combining features of a box plot and a kernel density estimate. The width of the violin at any given score level reflects the density of data points, offering insight into the variability and distribution of scores for each method. Overlaid within the violin are box plots, highlighting key summary statistics: the median (white line), the interquartile range (IQR) represented by the box, and whiskers indicating the range of scores within 1.5 times the IQR. Outliers beyond this range are also depicted, providing additional information on atypical performance.

The violin plot offers a clear comparison of the methods, showing differences in score consistency and variability. Methods with narrower and more symmetrical shapes, such as EigenGradCAM and ScoreCAM, indicate that scores are concentrated around a central value, suggesting stable and consistent performance across pathology classes. Conversely, methods with wider or asymmetrical distributions show a greater variability in ratings. This may reflect inconsistent reliability, where some pathology classes could benefit more from a given method than others. These visualizations complement the global averages and standard deviations presented in Table 1, allowing for a clearer interpretation of the general reliability and effectiveness of the methods. In the subsequent tables, the best method for each pathology is highlighted in bold.

**Table 1:** Global averages and standard deviations for each CAM method.

Method	Global average ± Standard deviation
AblationCAM	2.238 ± 1.287
EigenCAM	2.519 ± 1.228
EigenGradCAM	**2.571** ± **1.256**
FullGrad	2.000 ± 1.300
GradCAM	2.068 ± 1.178
GradCAM++	2.512 ± 1.277
GradCAM- ElementWise	2.160 ± 1.185
HiResCAM	2.179 ± 1.299
LayerCAM	2.460 ± 1.237
ScoreCAM	2.401 ± 1.438
XGradCAM	1.858 ± 1.198

#### ANOVA

**Fig. 4:**
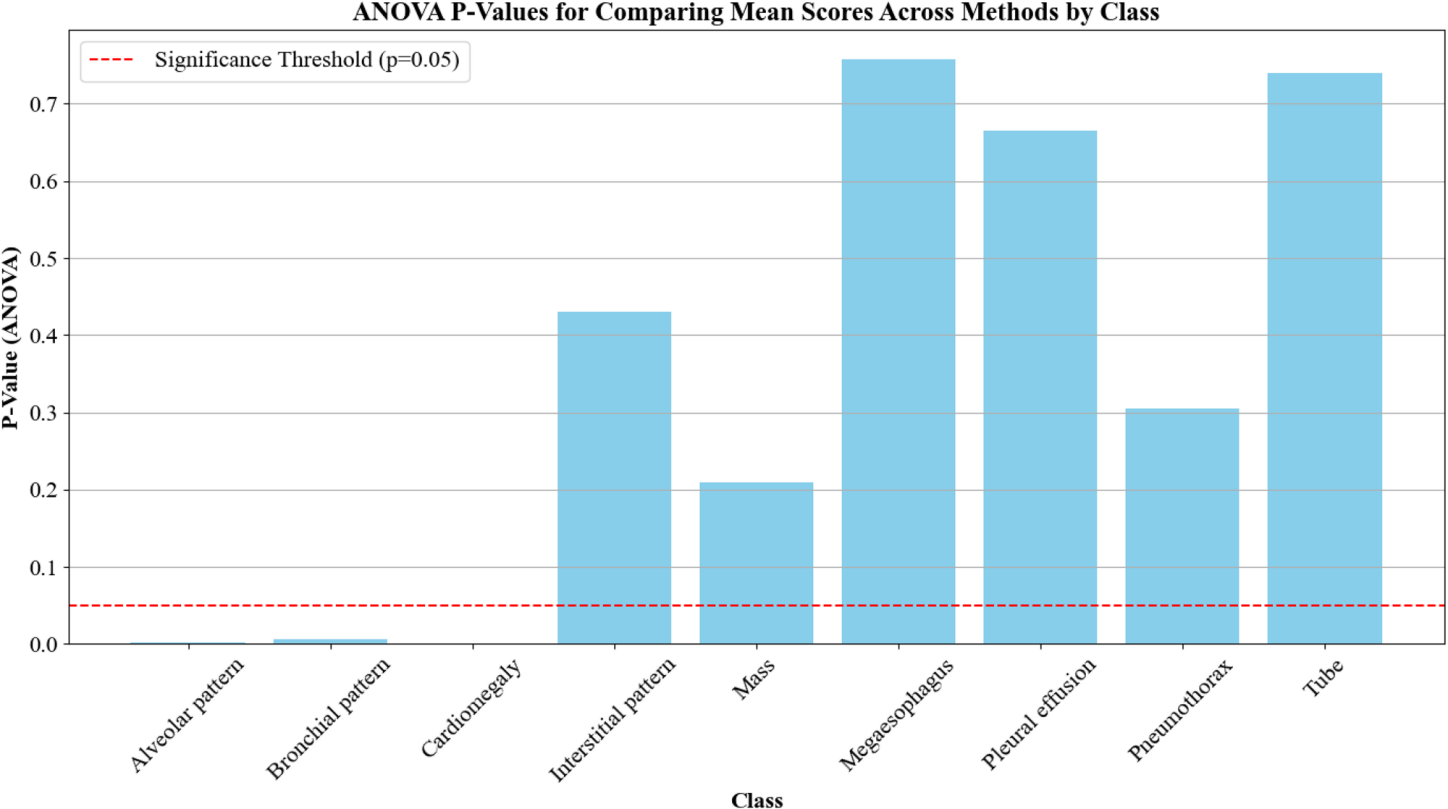
ANOVA test p-values for comparing mean scores across CAM methods by class. The red line shows the significance threshold (*p*=0.05).

The analysis of variance (ANOVA) test was performed to assess overall differences in mean scores between methods for each pathology class, the results of which can be seen in Fig. 4. A horizontal red dashed line was added to indicate the Significance Threshold of* p* = 0.05, where any result under would warrant rejection of the null hypothesis (no statistically significant difference in mean scores among the various CAM methods for a specific class).

Based on the results, significant differences were observed in the following classes: (i) Cardiomegaly: * p* = 0.00012, (ii) Alveolar pattern: * p* = 0.0014, (iii) Bronchial pattern: * p* = 0.0061. Therefore, considering that the ANOVA test yielded statistically significant results for these three classes, it would indicate that at least one method’s mean score differed significantly from the others. Furthermore, these findings suggest that the choice of the CAM method impacted the quality of interpretability in the said pathologies and, therefore, will be further evaluated.

#### Statistical analysis

To determine the statistical significance of the differences among the CAM methods, pairwise t-tests *with Benjamini–Hochberg false-discovery-rate (FDR) adjustment* were performed within each class. From these results, significant differences in several comparisons (FDR-adjusted $q < 0.05$) were identified only in the “Cardiomegaly”, “Alveolar pattern”, and “Bronchial pattern” classes. These classes align with those deemed significant through the ANOVA testing and thus are the focus of the detailed evaluation below. The mean explainability scores for the specific CAMs in each of these three pathologies are presented in Table 2.

To identify which methods differed significantly within each of the three pathologies, the FDR-adjusted pairwise t-tests were tabulated; statistically significant contrasts are shown in pathology-specific summary tables, while the full* p*-value matrices are provided in Additional Information Section A, in Figs. 12, 13, 14.

**Table 2:** Combined table of mean scores and standard deviations (SD) of each CAM method for the three selected pathologies.

Method	Cardiomegaly	Alveolar pattern	Bronchial pattern
AblationCAM	2.333 ± 1.042	2.278 ± 1.256	2.361 ± 1.334
EigenCAM	3.111 ± 1.282	3.250 ± 0.967	**3.167** ± **1.134**
EigenGradCAM	**3.306** ± **1.327**	3.000 ± 1.171	3.139 ± 1.099
FullGrad	1.611 ± 0.766	2.056 ± 1.218	1.861 ± 1.175
GradCAM	2.111 ± 1.063	2.111 ± 1.008	2.306 ± 1.470
GradCAM++	2.472 ± 1.108	2.500 ± 1.082	2.806 ± 1.142
GradCAMElementWise	2.417 ± 1.180	2.278 ± 1.386	2.222 ± 1.290
HiResCAM	2.667 ± 1.398	1.806 ± 1.091	2.444 ± 1.443
LayerCAM	2.361 ± 0.990	2.778 ± 1.290	2.944 ± 1.094
ScoreCAM	2.333 ± 1.121	**3.361** ± **1.073**	3.028 ± 1.230
XGradCAM	2.000 ± 1.014	1.472 ± 0.845	1.667 ± 1.014

#### Cardiomegaly

For the cardiomegaly class, the mean explainability scores for each CAM method are summarized in Table 2. Methods such as EigenGradCAM and EigenCAM achieved the highest mean scores of 3.306 (SD = 1.327) and 3.111 (SD = 1.282), respectively, while methods such as FullGrad and XGradCAM had lower mean scores of 1.611 (SD = 0.766) and 2.000 (SD = 1.014), respectively.

To identify which methods differed significantly, pairwise t-tests were performed between the methods; see Table 6. Significant differences (*p* < 0.05) were observed between EigenGradCAM and methods such as FullGrad (*p* = $0.000001$), GradCAM (*p* = 0.0015), and XGradCAM (*p* = 0.00036). Similarly, EigenCAM showed significant differences compared to FullGrad (*p* = $0.000006$), GradCAM (*p* = 0.008), and XGradCAM (*p* = 0.0022).

#### Alveolar pattern

In the alveolar pattern class, each method’s mean scores and standard deviations are presented in Table 2. ScoreCAM achieved the highest mean score of 3.361 (SD = 1.073), closely followed by EigenCAM with a mean score of 3.250 (SD = 0.967) and EigenGradCAM with a mean score of 3.000 (SD = 1.171). XGradCAM had the lowest mean score of 1.472 (SD = 0.845). An overall similarity to the Cardiomegaly class.

The pairwise t-tests in Table 7 showed that ScoreCAM significantly outperformed XGradCAM (*p* = $0.000000$), FullGrad (*p* = 0.00026), and GradCAM (*p* = 0.00011). Similarly, EigenCAM had significantly higher scores than XGradCAM (*p* = $0.000000$) and GradCAM (*p* = 0.00022). EigenGradCAM also performed significantly better than XGradCAM (*p* = 0.000002).

#### Bronchial pattern

Lastly, for the bronchial pattern class, the mean scores and standard deviations are shown in Table 2. Once again, similarly to the previous two classes, the EigenCAM and EigenGradCAM methods achieved the highest mean scores of 3.167 (SD = 1.134) and 3.139 (SD = 1.099), respectively. ScoreCAM also performed well, with a mean score of 3.028 (SD = 1.230). XGradCAM had the lowest mean score of 1.667 (SD = 1.014), while FullGrad scored 1.861 (SD = 1.175).

The pairwise t-tests in Table 8 revealed that EigenCAM and EigenGradCAM scored significantly higher than XGradCAM (*p* = 0.000006 and *p* = 0.000006, respectively) and FullGrad (*p* = 0.00027 and *p* = 0.00029, respectively). ScoreCAM also significantly outperformed XGradCAM (*p* = 0.00011) and FullGrad (*p* = 0.0021). There were significant differences between XGradCAM and other methods like LayerCAM (*p* = 0.00011) and GradCAM++ (*p* = 0.00065).

#### The remaining classes

The exact results of the remaining 6 classes can be seen in Table 3 , however due to high SD, very similar average scores and statistical insignificance (both in ANOVA and pair-wise t-test) towards a conclusion, they were not discussed in depth in this article. Furthermore, the exact results of the pair-wise t-test are provided in Additional Information Section A, in Figs. 15, 16, 17, 18, 19, 20. However, by looking at the previous classes, it is clear that a superior method might emerge.

**Table 3:** Combined table of mean scores and standard deviations (SD) of each CAM method for the six remaining pathologies.

Method	Interstitial pattern	Mass	Megaeso- phagus	Pleural effusion	Pneumothorax	Tube
AblationCAM	2.417 ± 1.296	1.167 ± 0.378	1.944 ± 1.530	2.417 ± 1.296	2.944 ± 1.351	2.278 ± 1.137
EigenCAM	2.472 ± 0.941	1.861 ± 1.199	1.472 ± 0.774	2.222 ± 1.198	2.528 ± 1.134	**2.583** ± **1.180**
EigenGradCAM	**2.944** ± **1.218**	1.583 ± 0.732	1.500 ± 0.775	2.472 ± 1.000	2.667 ± 1.394	2.528 ± 1.108
FullGrad	1.806 ± 1.191	1.306 ± 0.525	**2.000** ± **1.352**	1.833 ± 1.108	**2.972** ± **1.732**	2.556 ± 1.539
GradCAM	2.333 ± 1.287	1.333 ± 0.586	1.417 ± 0.770	2.250 ± 0.996	2.694 ± 1.261	2.056 ± 1.330
GradCAM++	2.667 ± 1.434	**2.333** ± **1.604**	1.944 ± 1.372	**2.639** ± **1.199**	2.833 ± 1.183	2.417 ± 1.180
GradCAM- ElementWise	2.222 ± 1.245	1.472 ± 0.609	1.611 ± 0.903	2.306 ± 1.037	2.611 ± 1.293	2.306 ± 1.167
HiResCAM	2.306 ± 1.411	1.417 ± 0.692	1.639 ± 0.867	2.417 ± 1.296	2.722 ± 1.365	2.278 ± 1.386
LayerCAM	2.556 ± 1.229	2.000 ± 1.309	1.611 ± 0.964	2.528 ± 1.444	**2.972** ± **1.158**	2.389 ± 1.050
ScoreCAM	2.222 ± 1.333	2.056 ± 1.672	1.972 ± 1.558	1.861 ± 1.291	2.611 ± 1.400	2.167 ± 1.558
XGradCAM	2.111 ± 1.326	1.417 ± 1.025	1.778 ± 1.333	2.333 ± 1.512	2.028 ± 1.230	1.917 ± 1.156

#### Annotator agreement

**Fig. 5:**
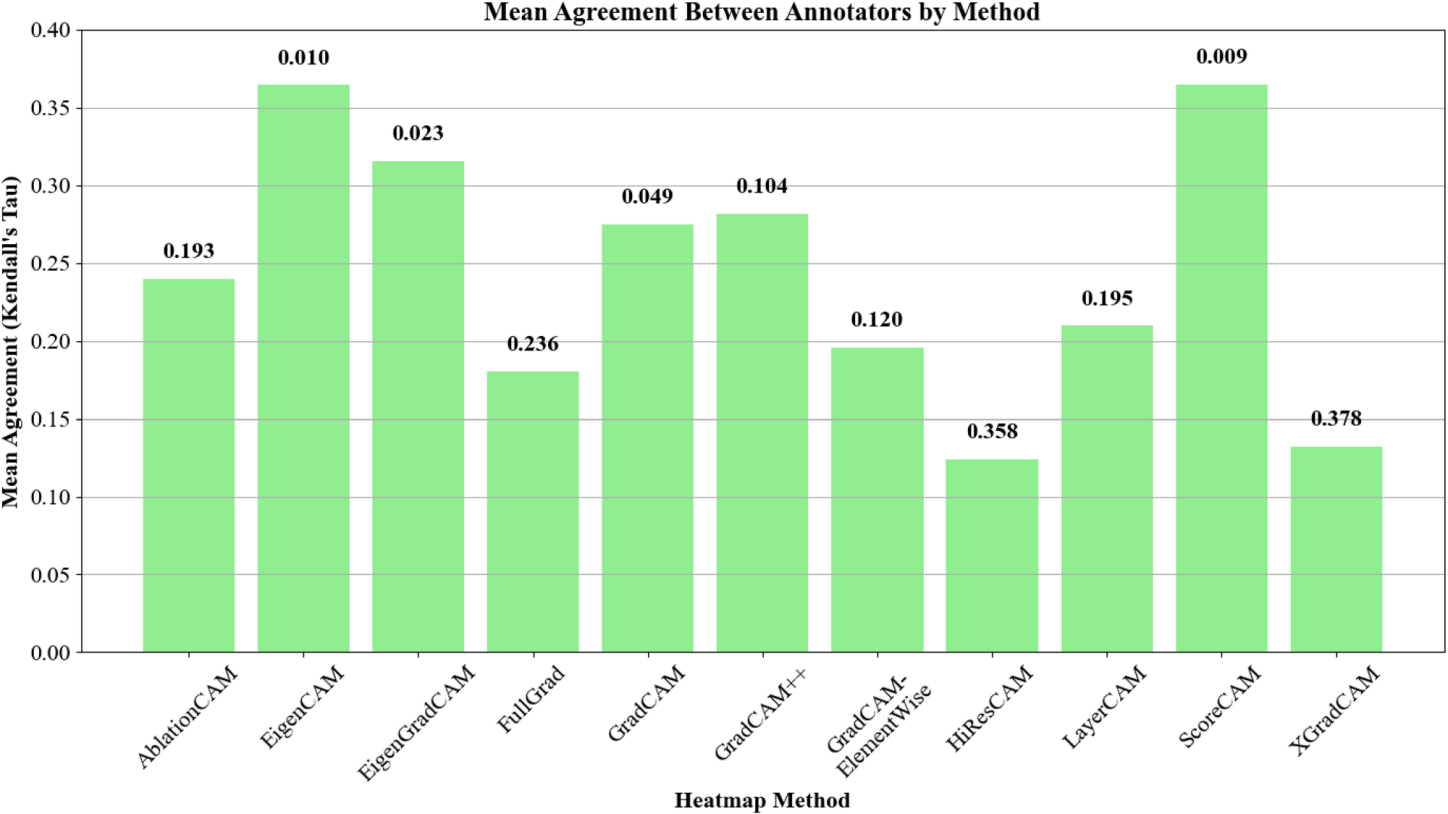
Agreement between annotators by CAM method, measured using Kendall rank correlation coefficient (Kendall’s Tau coefficient).

In addition, the consistency of the evaluations between the annotators was measured using the Kendall rank correlation coefficient, of which the results can be seen in Fig. 5, with p-values for each class being indicated above each bar. Methods such as ScoreCAM (mean agreement = 0.365,* p* = 0.009), EigenCAM (mean agreement = 0.364,* p* = 0.010), and EigenGradCAM (mean agreement = 0.316,* p* = 0.023) exhibited the greatest agreement among the annotators. This indicates that these methods produced more consistently interpretable results as judged by different veterinary experts.

### Absolute scores: best performing

The second part of the survey aimed to identify the best CAM method from each set, which outperformed the rest of the CAMs in the aspects of localization and color. The selected methods were then graded on an absolute scale of 1-5, with 1 indicating the poorest performance and 5 representing the optimal heatmap coverage. Importantly, this evaluation allowed for different methods to be chosen as the best-performing representation for localization and color metrics.

#### Overall ranking

**Fig. 6:**
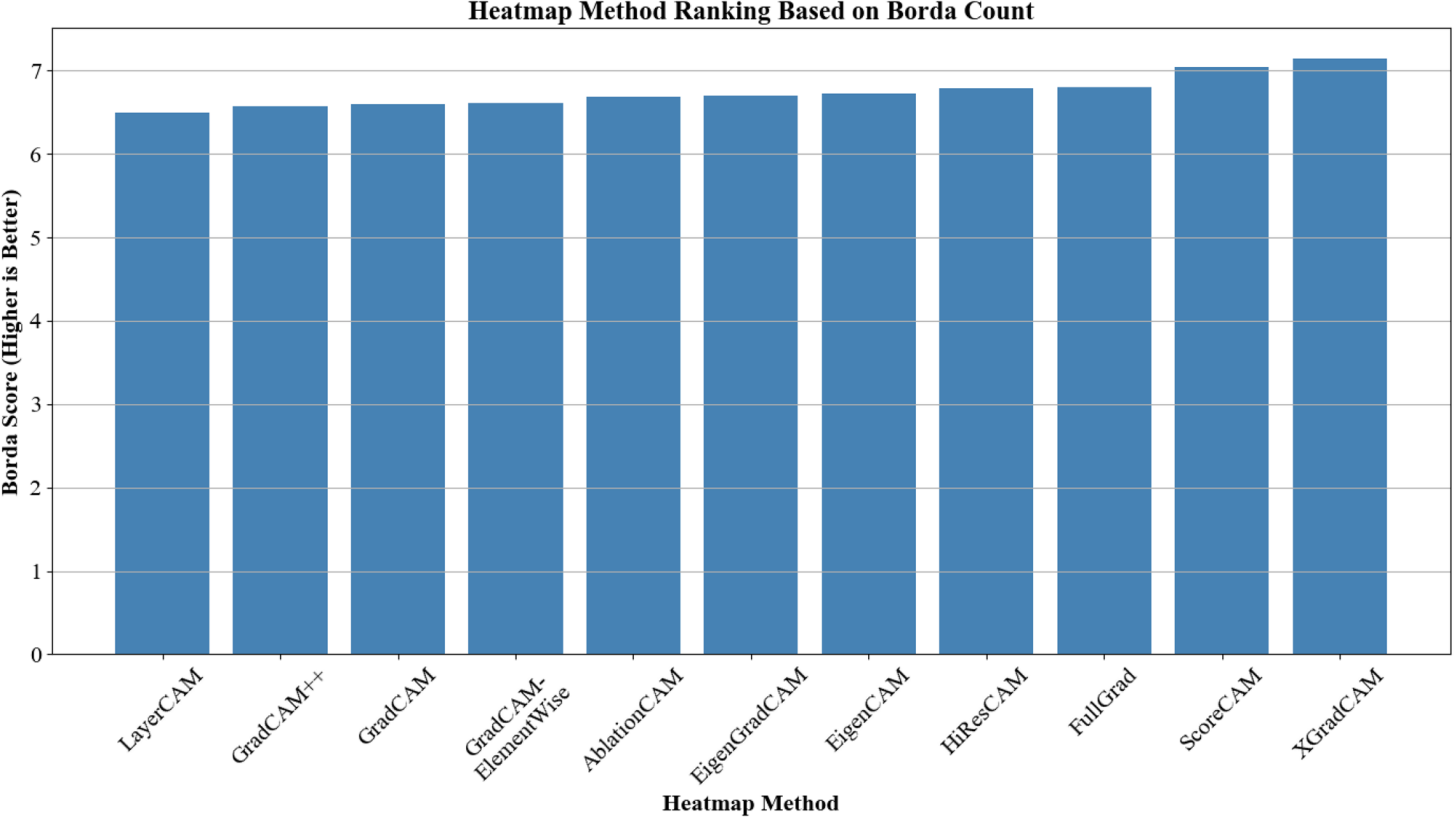
Borda count for each respective method, determined by how often it is selected as the Best Performing option among all methods within a specific image set.

Based on the absolute scale scores, the Borda count method established an overall ranking of the CAM methods, combining performance scores across color and localization. The results are presented in Fig. 6 with the scores of top-ranked methods as follows: XGradCAM (Borda score = 7.148), ScoreCAM (Borda score = 7.037), HiResCAM (Borda score = 6.796).

In particular, since higher Borda scores indicate better rankings, it would suggest that XGradCAM provides a balanced and practical approach to explainability in veterinary radiography, closely followed by ScoreCAM and HiResCAM. However, with the maximum score obtainable based on nine classes and three respondents being 24, none of the methods achieved more than 30%, relative to the maximum score. This suggests that the highest-scoring methods received a mix of rankings and were not consistently ranked at the top. Therefore, while methods such as XGradCAM and ScoreCAM had higher scores, the differences and the results themselves were not substantial enough to determine whether they significantly influenced clinical decisions.

#### Best performing methods

**Fig. 7:**
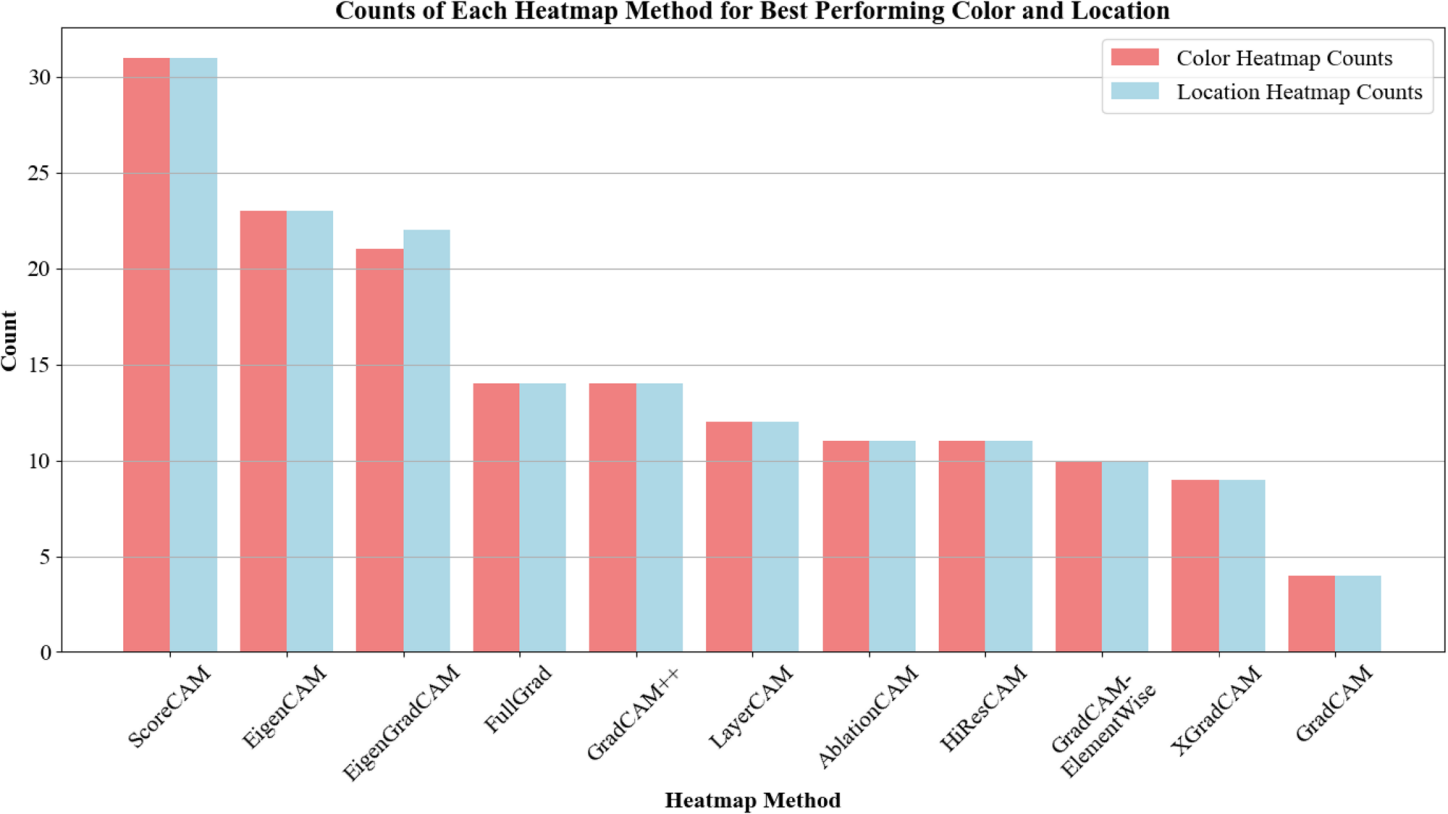
Frequency of selection for methods in the Best Performing category (out of 11 evaluated).

In addition, the annotators identified the best-performing methods based on their color and localization evaluations. ScoreCAM was observed most frequently as the method that performed the best in both categories, with 31 counts each, as seen in Fig. 7. EigenCAM and EigenGradCAM also received high counts, reinforcing their effectiveness in generating interpretable and clinically relevant heatmaps.

Furthermore, when considering both the frequency of a method being selected as best performing and the Borda count scores, it becomes evident (with the exception of ScoreCAM) that methods which were awarded a higher score were, in fact, selected less often as best performing compared to those with mediocre and lower scores.

**Fig. 8:**
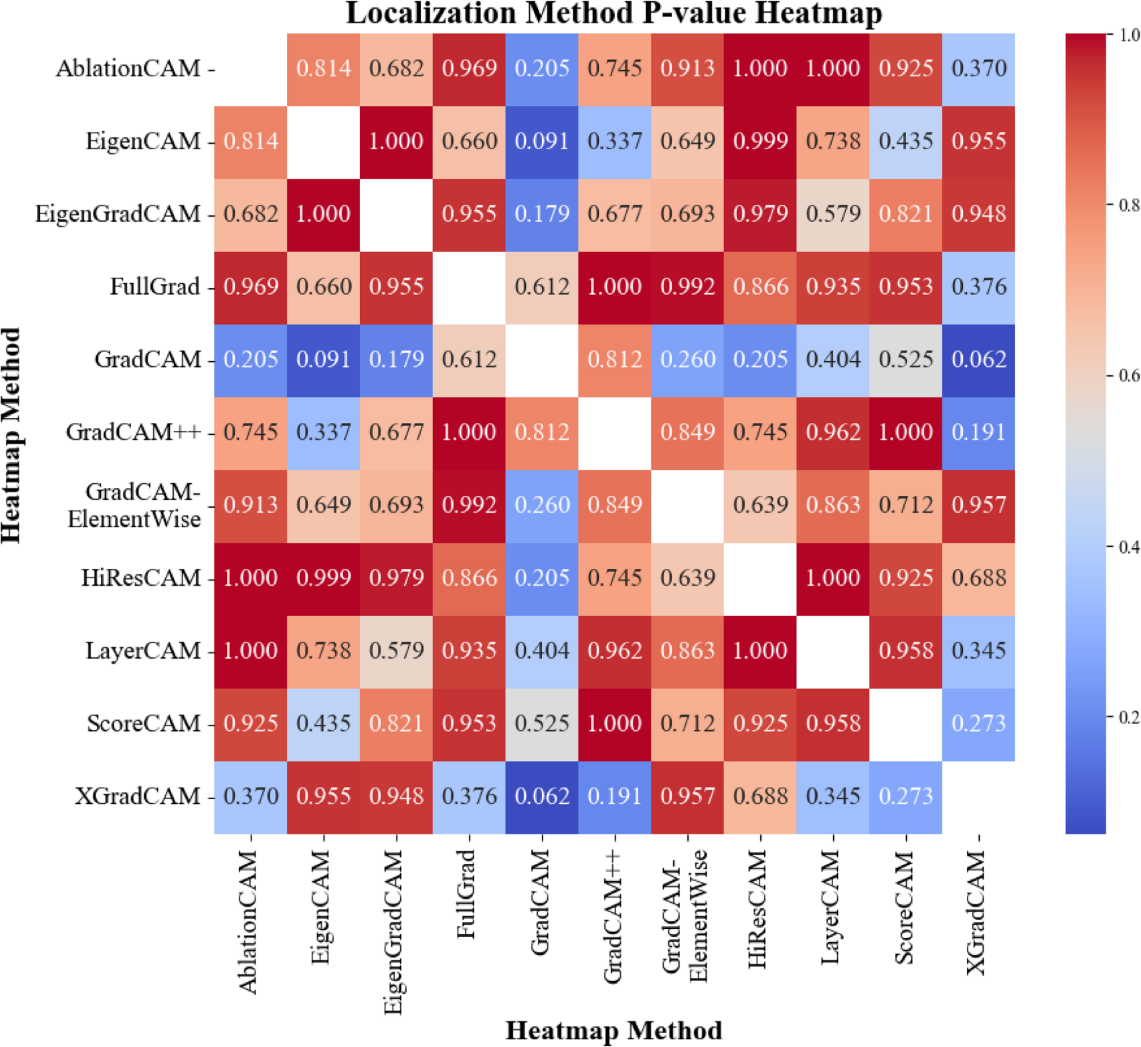
*p*-values derived from pairwise t-tests assessing localization metric scores among the Best Performing methods.

**Fig. 9:**
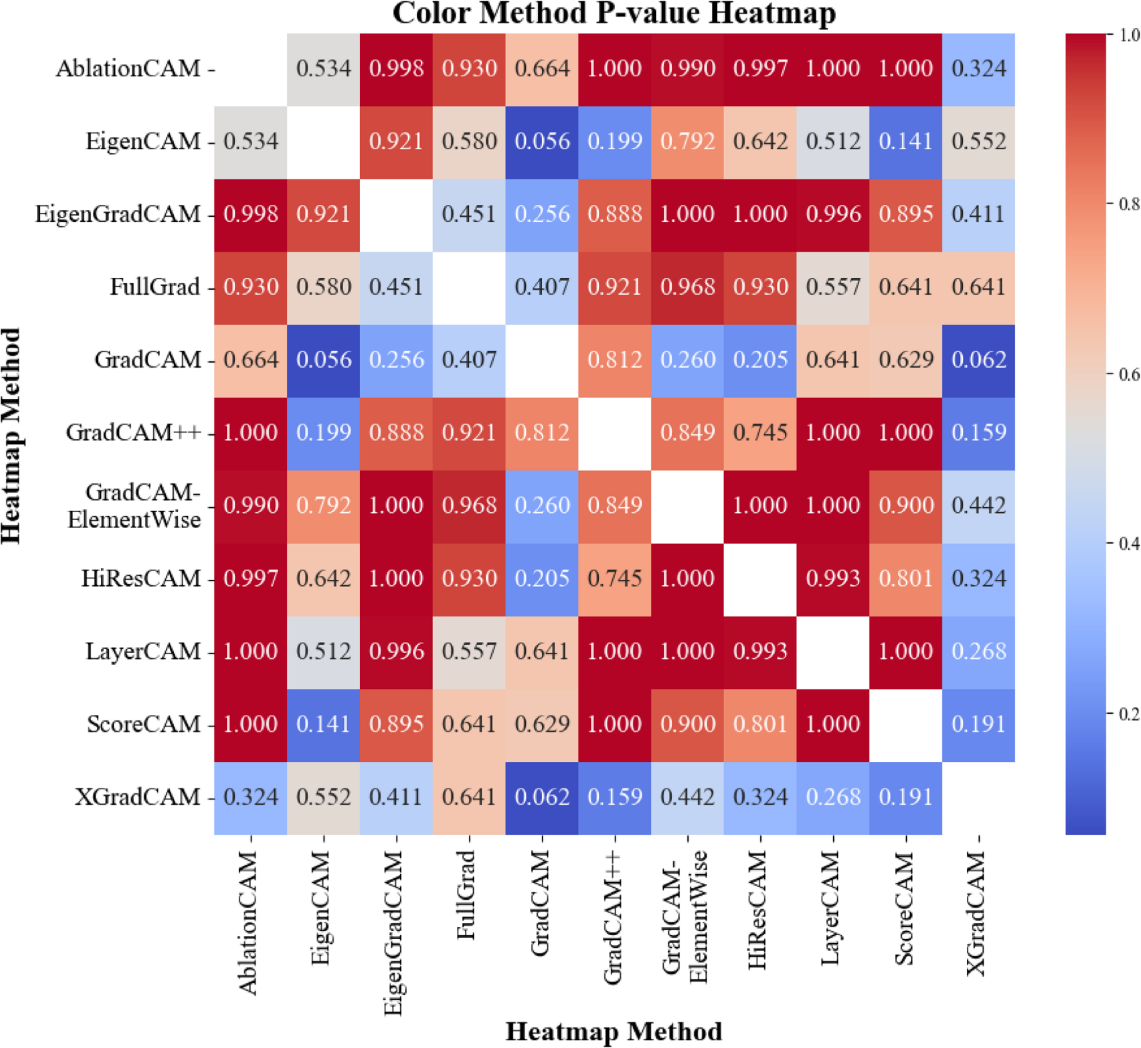
*p*-values derived from pairwise t-tests assessing colour metric scores among the Best Performing methods.

#### Distribution analysis

Kolmogorov-Smirnov tests were performed to compare the distributions of the scores between the best methods selected (from the perspective of veterinarians). The results of the tests are shown in Figs. 8, 9 representing the best color distribution and localization accuracy, respectively. In both cases, most of the comparisons yielded high *p*-values, indicating that there were no significant statistical differences in the distribution of scores between most of the methods. This is to say that high p-values suggest that the two methods are similar, implying that neither is superior to the other. However, XGradCAM comparisons often resulted in lower *p*-values, implying that its score distribution differed from other methods in some instances. In addition, considering the frequency with which veterinarians choose this method as the best method suggests a statistically worse performance of the method compared to others.

## Discussion

This study sought to evaluate whether a range of CAM methods could enhance the explainability of CNN outputs in veterinary radiography. Although deep learning techniques have shown promise in improving the speed and accuracy of diagnoses, their “black box” nature continues to deter clinical adoption, especially in veterinary medicine, where explainability is crucial for building trust. Recognizing the challenges in bridging complex AI models and practical, clinician-friendly insights, we explored whether specific CAM techniques offer veterinarians clearer, more actionable visual explanations. The primary objective was to determine whether these methods can effectively support veterinarians in understanding AI-generated results and their diagnostic confidence in interpreting X-ray imagery.

Our initial observations indicated that the color patterns of the generated heatmaps often coincided with the expected pathological regions. This general alignment suggests that the proposed differentiation between the localization of the heatmap and its relative color intensity was unnecessary, as veterinarians do not perceive such differences. Furthermore, when we examined the subjective perceptions of veterinarians, no single CAM method clearly outperformed the others. In other words, while visual representations were directionally correct, their actual impact on improving veterinarians’ understanding and confidence in AI-assisted diagnoses appeared limited.

To quantify these perceptions, we performed analysis of variance (ANOVA) tests for each pathology class. For most classes, including the “Interstitial pattern” (*p* = 0.430), “Mass” (*p* = 0.210), “Megaesophagus” (*p* = 0.759), “Pleural effusion” (*p* = 0.666), “Pneumothorax” (*p* = 0.304), and “Tube” (*p* = 0.741), the high *p*-values indicated that any observed differences between CAM methods were not statistically significant. This suggests that from the perspective of practicing veterinarians, switching from one CAM approach to another did not significantly improve the explainability of CNN-derived insights.

Although a few classes–such as “Cardiomegaly” (*p* = 0.00012), “Alveolar pattern” (*p* = 0.0014), and “Bronchial pattern” (*p* = 0.0061)–did show statistically significant differences, the practical implications on mean scores were modest. For example, while EigenGradCAM outscored XGradCAM in the class “Cardiomegaly” (3.306 vs. 2.000 on a scale of 1–5), both methods ultimately yielded only moderate levels of perceived usefulness. Thus, despite some marginal variations, none of the examined CAM techniques consistently provided the level of transparency and reassurance veterinarians would need to fully integrate these tools into their diagnostic workflows.

Moreover, the lack of statistically significant differences across most pathology classes would imply that, from the veterinarians’ perspective, the choice of the CAM method does not substantially affect the explainability of the CNN outputs in a clinical setting. Although methods such as ScoreCAM and EigenCAM were among the most frequently indicated as the best performing by the annotators (Fig. 7), with respective counts of 31 and 23, perceived usefulness for all eleven CAM methods remained moderate as their mean scores fell in a narrow band between 2 and 3 on a 5-point Likert scale. In other words, even the preferred methods did not meet clinicians’ expectations for clear, decision-shaping explanations, limiting interpretability and clinical utility. Indicating that while some CAM methods may be preferred for specific use cases, no single method universally excelled across all pathology classes. The Borda count ranking, further highlighted that even the top-ranked methods did not significantly outperform others in a clinically meaningful way (Fig. 6). Interestingly, these differences in visual explanations seem to hinge on the class in question. For certain classes, the choice of the CAM method barely changes the interpretation, while for others, it can lead to noticeably different activation maps that might affect the trust or use of these explanations.

Furthermore, the clinical weight given to heat-map precision is far from uniform across thoracic pathologies. For lesions whose differential diagnosis is strongly tied to where they occur, such as interstitial, alveolar and bronchial infiltrates, small spatial shifts in an activation map can redirect a veterinarian from a correct diagnosis, and therefore alter treatment plans in a negative way. In contrast, for predominantly global conditions such as cardiomegaly or pneumothorax, coarse highlighting of the cardiac silhouette or pleural margin often suffices. These observations imply that evaluation frameworks for veterinary XAI should prioritize localization errors based on their potential clinical impact instead of using a single threshold for all pathology classes. This also clarifies why the veterinarians in our study more severely penalized CAM disagreements in infiltrative patterns compared to volumetric diseases.

There is no one-size-fits-all CAM solution. Some methods may be better for classes where the model’s decision hinges on a small, subtle detail, while others might work fine for classes where the key features are large and obvious. This aligns with our introduction’s emphasis on the importance of explainability methods, underlining that for reliable explanations from CAMs, we must consider both the type of CAM and the nature of the classes we are dealing with. However, doing so is unrealistic for practical situations where we strive for universal methods. Veterinarians, like human doctors, require precise and actionable explanations in every scenario to effectively integrate AI models into their diagnostic workflows, even before pathological classification.

Given the limitations observed, it suggests a limited differentiation among CAM methods and that if more robust and universal explanations are desired, future efforts may need to move beyond CAMs. Instead, exploring alternative XAI techniques, for example, concept-based techniques such as TCAV that quantify how user-defined clinical concepts drive predictions, self-attention models whose intrinsic attention maps can be read as fine-grained localization cues, and prototype-based networks that justify decisions by comparing an image with learned canonical cases. Although these methods promise explanations that are more in line with practical veterinarian needs, they also introduce additional conceptual and computational complexity that may be more difficult for clinicians to grasp and trust without targeted training. Clearer, clinically relevant insights must therefore be balanced against the increased learning curve if they are to enhance decision-making and adoption in everyday practice.

Several factors may have contributed to the moderate performance of the evaluated CAM methods. First, the limited resolution of their heatmaps, even after upscaling, generally results in a rough localization of the pathologies. This is because GradCAM-family methods down-sample feature information to the final 7 $\times$ 7 convolutional grid in ResNet-18 and then up-sample, so subtle nodules or bronchial thickening are inevitably blurred. Second, the complexity of veterinary imaging, given that diverse anatomical structures and variations across breeds make it harder for CAMs to generalize effectively. This is evident for example in thoracic conformation as it differs markedly between brachycephalic, barrel-chested, and deep-chested dogs, altering heart silhouette, rib curvature, and lung-field geometry. Third, limited semantic understanding as saliency maps are fundamentally gradient- or activation-weighted textures; they do not encode higher-level concepts such as “left caudal lung lobe” or “pleural line”. Although newer concept-based or causal-probe hybrids promise richer explanations, these approaches remain experimental. Fourth, the subjectivity of responders, as relying on human annotators introduces personal biases. Inter-rater reliability among the three evaluators was only moderate: Kendall Tau values ranged from 0.12 to 0.38 across the eleven CAM methods, below the $\approx$ 0.50 threshold generally interpreted as “strong” agreement. Moderate Tau is common when complex visual tasks are scored on Likert scales by small expert panels because the sampling variance of Tau grows rapidly as N decreases, and because ordinal scales compress the true dispersion of opinions. Lastly, our analysis focused solely on the ResNet-18 architecture, and different CNN models may interact differently with CAM methods.

Therefore, future research should explore hybrid strategies that embed semantic segmentation masks within the explainer pipeline, couple radiographs with complementary clinical data through multimodal XAI frameworks, and tailor explanation modules to the visual signatures of individual pathologies. Such targeted advances offer a more promising route to trustworthy, clinically actionable AI support and ultimately to better patient outcomes in veterinary practice. Additionally, future studies should recruit larger and more diverse annotator cohorts or employ consensus protocols (e.g., Delphi rounds) to strengthen reliability.

*Conclusion* In conclusion, while CAM methods such as ScoreCAM and EigenCAM are among the more informative choices, their benefits remained modest and inconsistent, confirming that CAM methods, at least in their current form, are insufficient for reliable, day-to-day integration in veterinary radiology. The absence of statistically significant, confidence-boosting differences across the eleven techniques, as perceived by veterinarians, underscores a broader limitation of gradient-based saliency: they seldom deliver the clear, anatomy-aligned explanations clinicians require at the point of care. Therefore, exploring alternative or supplementary explainability methods is crucial to enhance the integration of AI models into veterinary diagnostics.

## Methods

### Flowchart

**Fig. 10:**
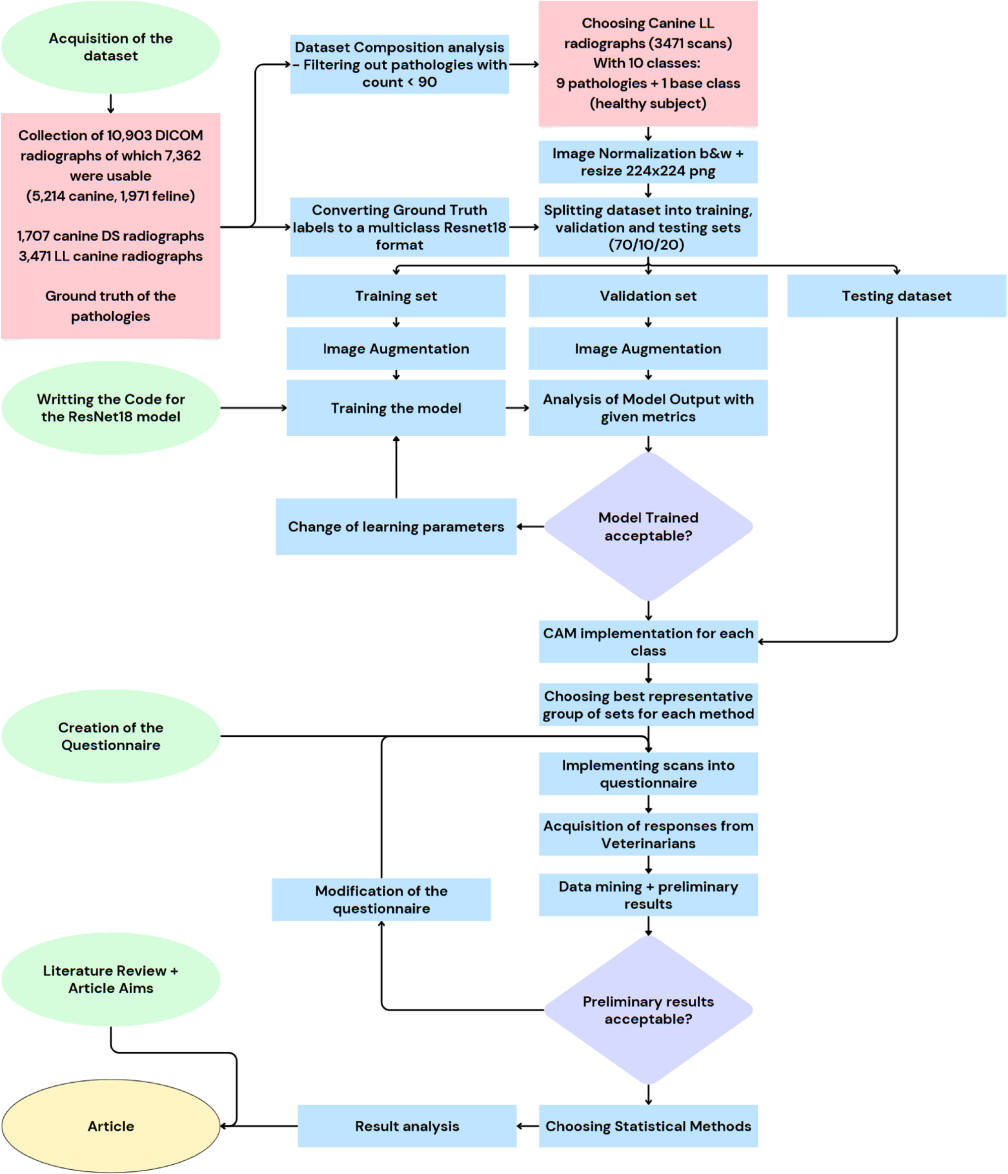
Flowchart of the Methodology Section of this article.

The flowchart shown in Fig. 10 outlines the key steps of the methodology. It starts with acquiring and preparing the data set, including filtering and splitting the data into training, validation, and testing sets. The ResNet-18 model was then implemented and trained with image normalization and augmentation. After training, CAM methods were applied to generate heatmaps for each class, which were incorporated into a questionnaire for veterinarians to assess. The responses were statistically analyzed to assess the explainability, which formed the basis for the study’s conclusions.

### Dataset

The dataset made available for this study consisted of 10903 DICOM canine and feline radiographs, of which 7362 were usable images, which further consisted of 5214 canine and 1971 feline. In addition, the canine group was composed of 3471 latero-lateral (LL) and 1707 dorso-ventral (sagittal)(DV) radiographs. Based on the group size, only canine LL projections were chosen for this study as their differentiation was the greatest. The number of radiographs that contained each pathology (later referred to as class / pathology) is listed in Table 4 below and is based on ground truth evaluation conducted and provided by veterinarians at the Veterinary Teaching Hospital of the University of Padua. It is important to note that this study being a multilabel problem, it was common for several tags to be present in one radiograph; thus, the total number of tags exceeded the total number of radiographs present in the dataset. All pathologies with an occurrence count of more than 90 were included in the final dataset, which included training, validation, and test sets. The dataset was subsequently divided into training, validation, and test sets, following a standard split of 70%, 10%, and 20%, respectively, which is a common practice in machine learning to ensure a correct evaluation of the model^33^.

**Fig. 11:**
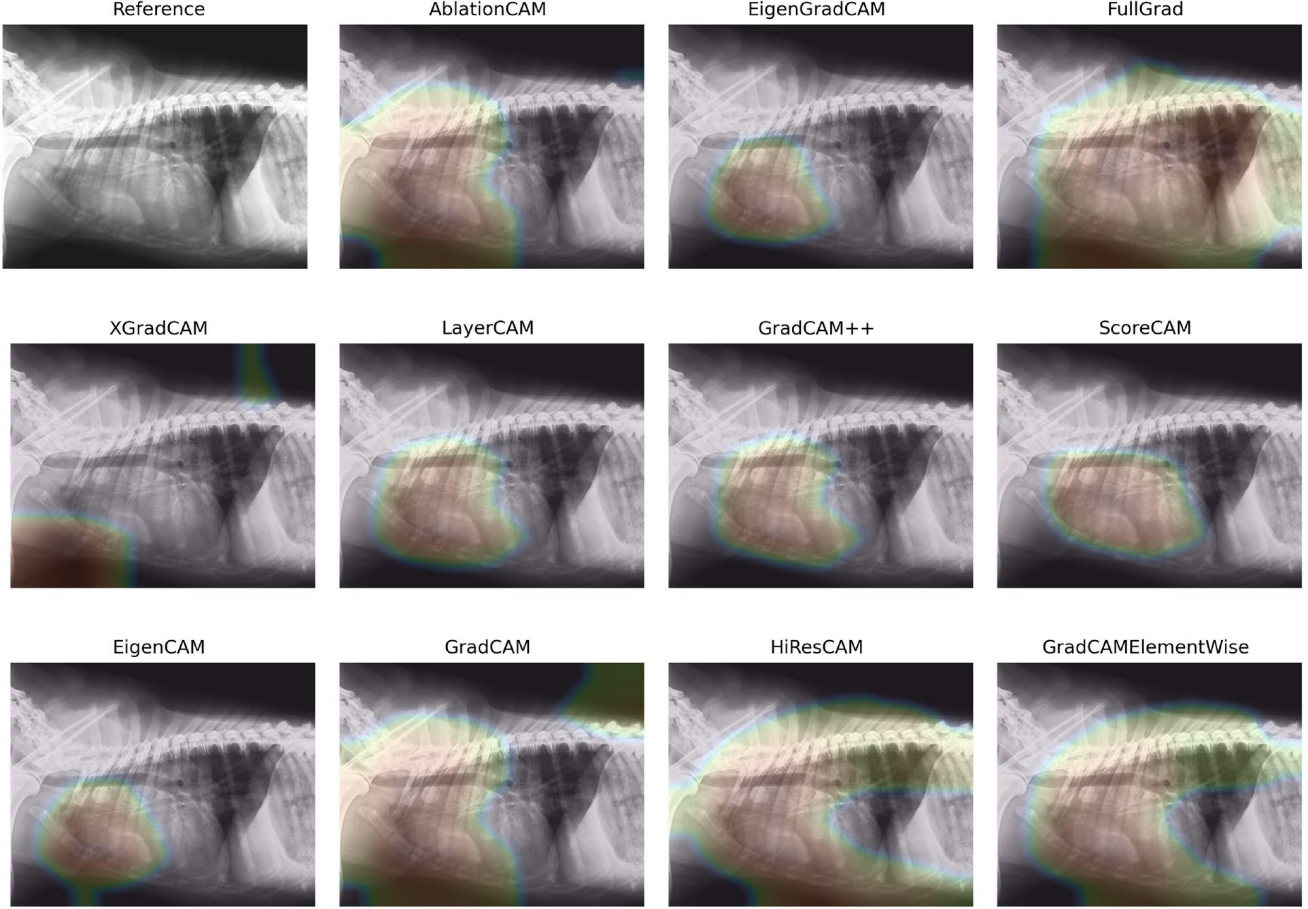
Example radiograph from the Alveolar Pattern class, with the corresponding eleven CAM methods.

**Fig. 12:**
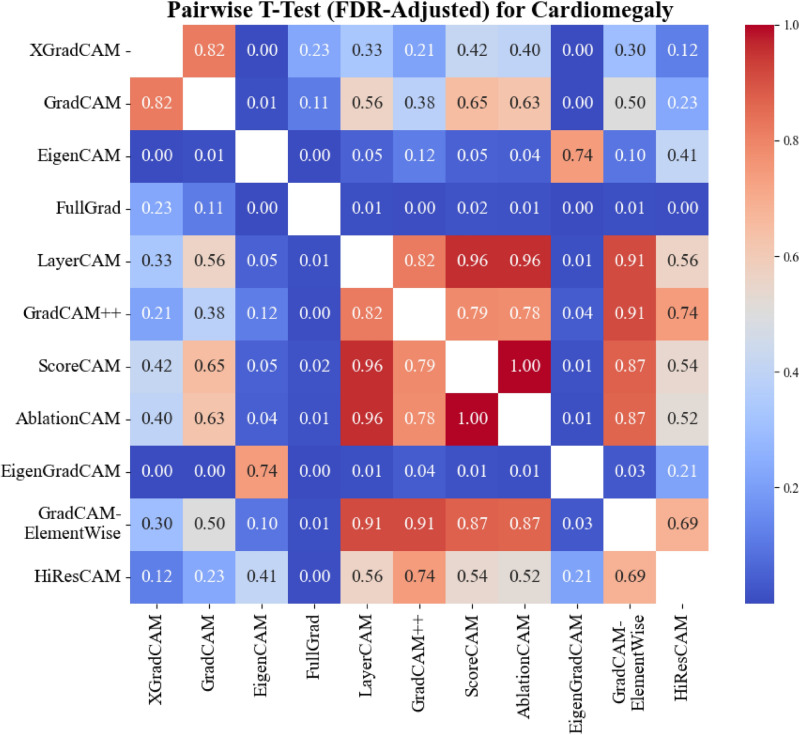
p-values derived from pairwise t-tests assessing average scores among the methods in Cardiomegaly pathology.

**Fig. 13:**
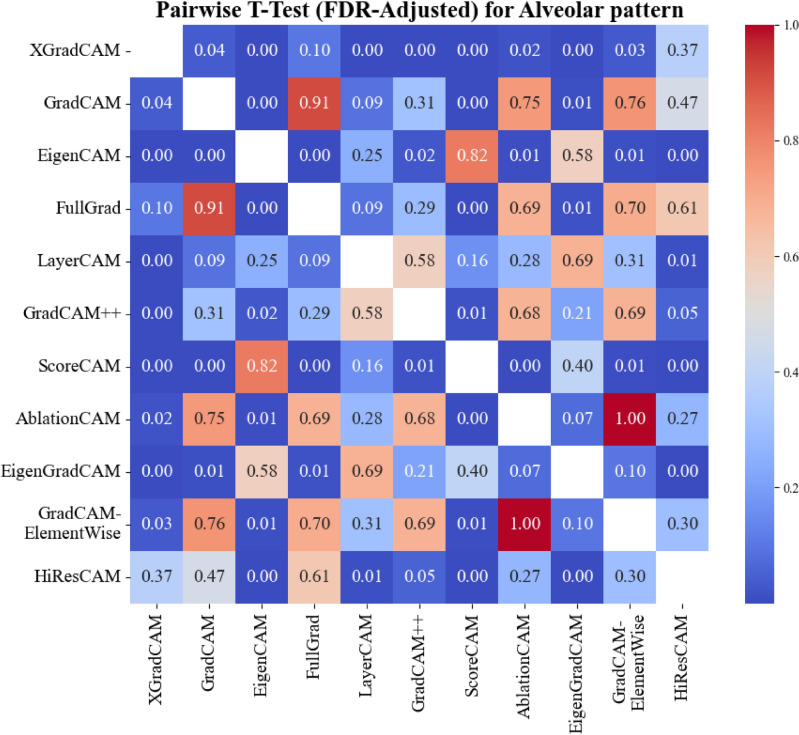
*p*-values derived from pairwise t-tests assessing average scores among the methods in Alveolar pattern pathology.

**Fig. 14:**
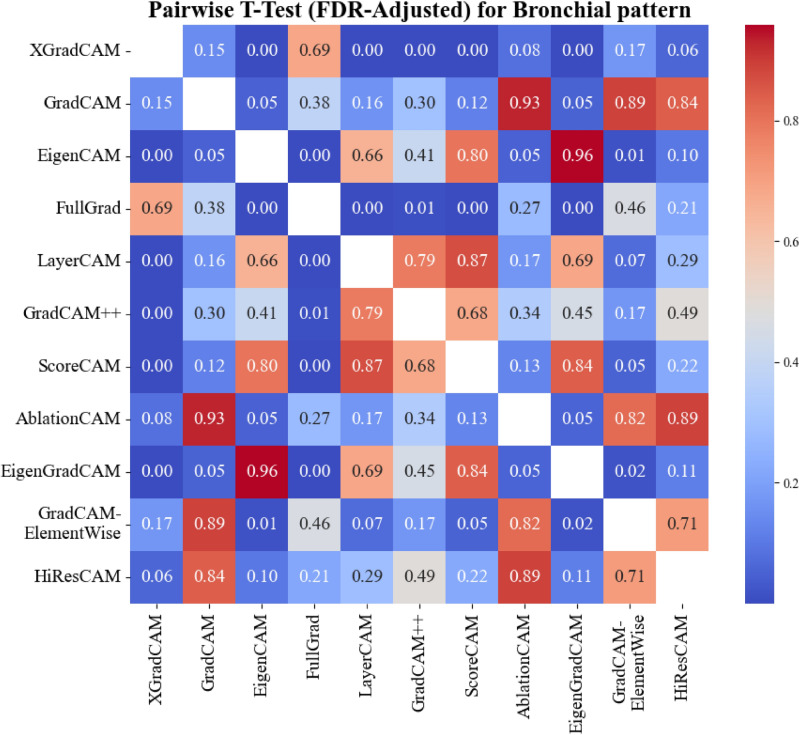
*p*-values derived from pairwise t-tests assessing average scores among the methods in Bronchial pattern pathology.

**Fig. 15:**
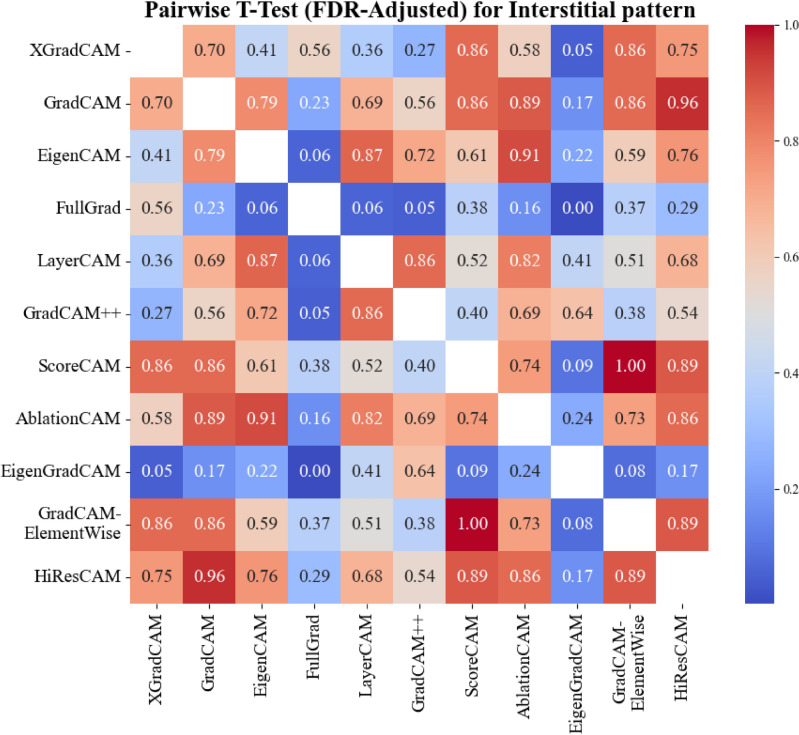
*p*-values derived from pairwise t-tests assessing average scores among the methods in Interstitial pattern pathology.

**Fig. 16:**
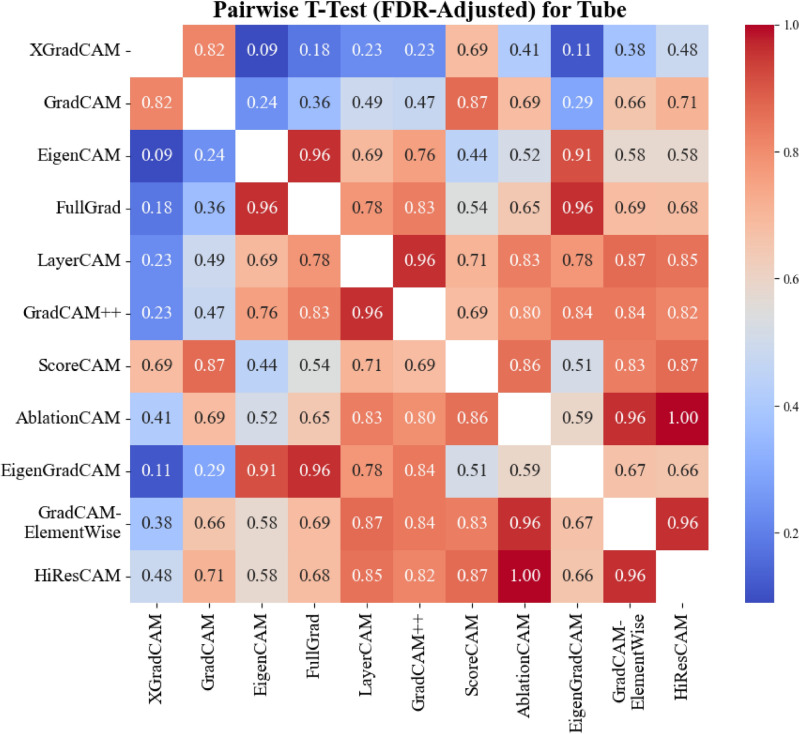
*p*-values derived from pairwise t-tests assessing average scores among the methods in Tube pathology.

**Fig. 17:**
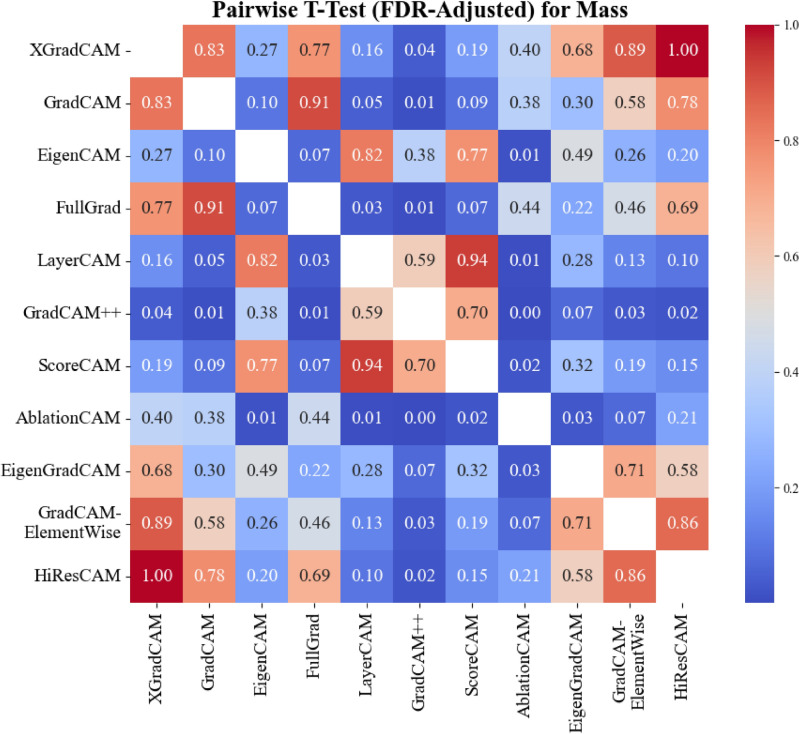
*p*-values derived from pairwise t-tests assessing average scores among the methods in Mass pathology.

**Fig. 18:**
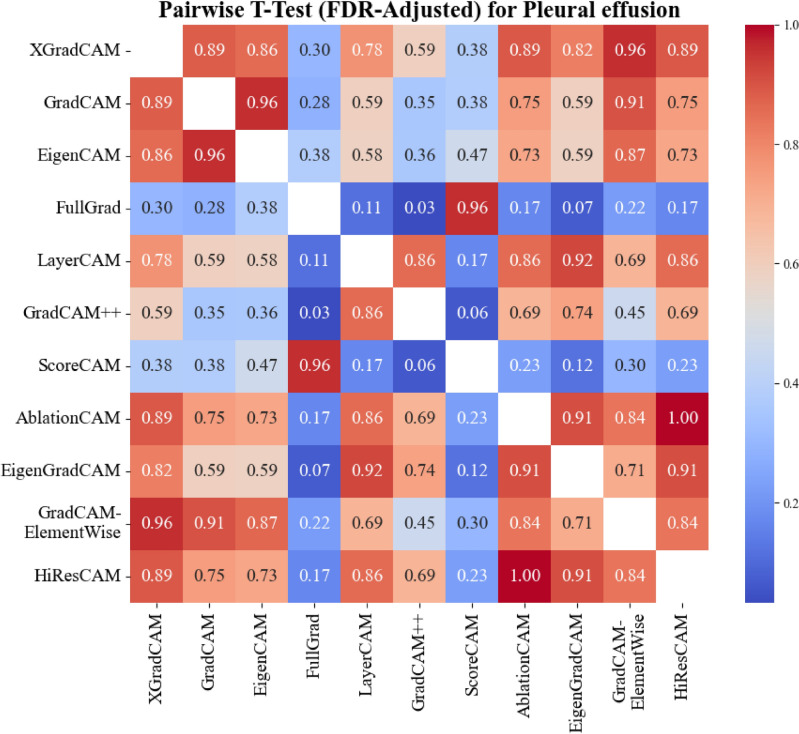
*p*-values derived from pairwise t-tests assessing average scores among the methods in Pleural effusion pathology.

**Fig. 19:**
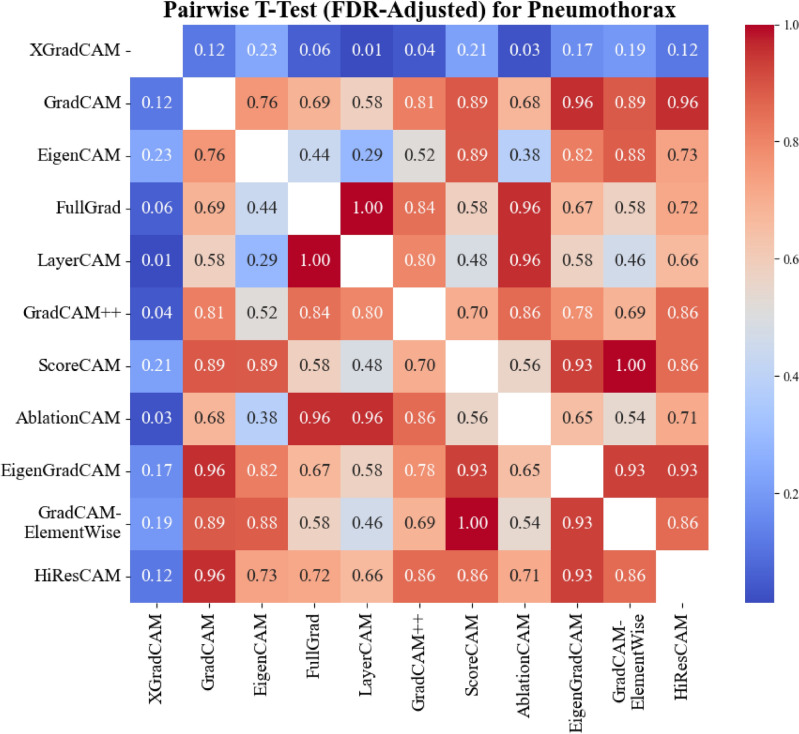
*p*-values derived from pairwise t-tests assessing average scores among the methods in Pneumothorax pathology.

**Fig. 20:**
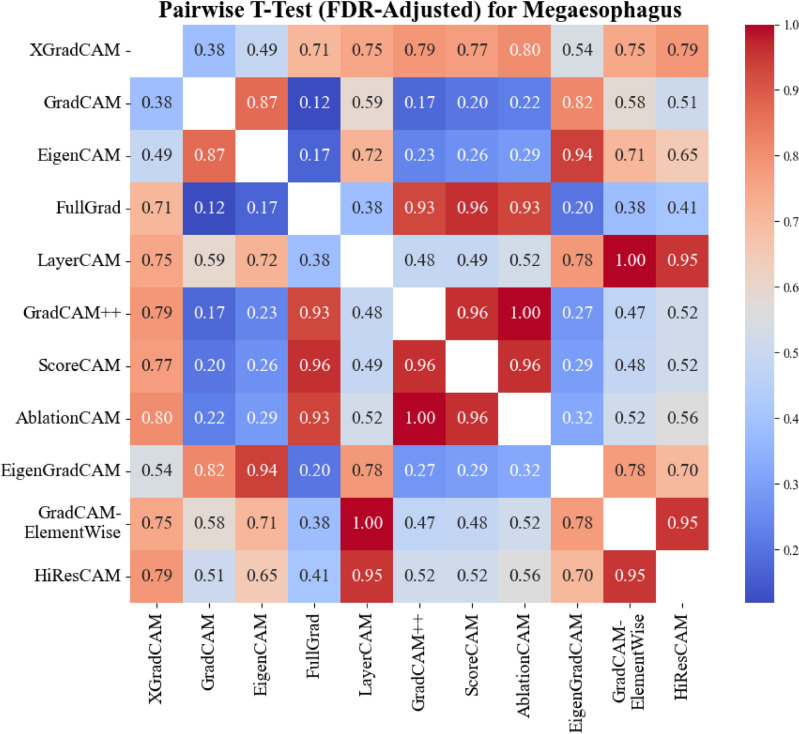
*p*-values derived from pairwise t-tests assessing average scores among the methods in Megaesophagus pathology.

Figure 11 represents heatmaps generated by each of the eleven CAM methods, further described in Table 5, in the context of the Alveolar Pattern pathology.

**Table 4:** Distribution of pathology types in dogs as marked by veterinarians.

Pathology types in dogs	Count
Not found	1760
Cardiomegaly	689
Interstitial pattern	478
Alveolar pattern	478
Bronchial pattern	443
Tube	338
Mass	169
Pleural effusion	115
Pneumothorax	97
Megaesophagus	95

### Models

The selection of a deep learning model for this research was made by assessing the performance of three prominent convolutional neural network (CNN) architectures (ResNet18^34^, ResNet50^34^, and EfficientNetV2^35^) based on their accuracy in classifying canine LL radiographs. The ResNet18 emerged as the best-performing architecture for the given real-life veterinary dataset, demonstrating a superior overall accuracy compared to its counterparts. This is further supported by existing literature highlighting the effectiveness of residual network (ResNet) architectures in image classification tasks^36–38^.

The following benchmark works on the basis of implementing a multilabel model which, when provided with a canine LL X-Ray scan as an input, outputs probabilities of occurrence for all 10 classes (including 9 pathologies and 1 base class for healthy subjects). Each DICOM image of an X-ray scan is first converted to a black-and-white image, resized to 224x224 pixels, and normalized. The normalization process includes adjusting the pixel intensity values to the range [0, 1], rescaling them to [0, 255], and applying a brightness normalization factor. Moreover, extreme pixel intensities are moderated by substituting the maximum values with the second-highest intensity to reduce the impact of outliers before the image is loaded into the model.

All experiments were conducted in PyTorch (v 2.1) on a single NVIDIA V100 GPU, with $torch.manual\_seed(42)$ set for reproducibility. The ImageNet-pre-trained ResNet-18 backbone was fine-tuned using a gradual unfreezing schedule, progressively unlocking blocks layer4 $\rightarrow$ layer3 $\rightarrow$ layer2 $\rightarrow$ layer1, a strategy shown to stabilise transfer-learning convergence. Training ran for up to 120 epochs with a batch size of 64, the AdamW optimizer ($\beta _1$ = 0.9, $\beta _2$ = 0.999, $weight\_decay=0.01$) and an initial learning rate of $1 \times 10^{-4}$. The class imbalance was handled using BCEWithLogitsLoss and a ten-element $pos\_weight$ vector proportional to the prevalence of the pathology. Model selection relied on the highest macro F1-score on a stratified validation set, while early stopping (patience = 5 epochs without validation-loss improvement) curtailed over-fitting, and the best checkpoint was retained for saliency analysis.

### Data augmentation

Image augmentation was performed to further improve the overall accuracy of the model. This involved applying various transformations to the images during the loading process, ensuring that each augmentation pattern was unique for each training iteration. It is crucial to note that this was achieved by adjusting each augmentation’s non-zero probability of occurrence separately based on the severity of image distortion it caused, reducing the likelihood of losing meaningful data within the image while still improving the model’s ability to generalize across different representations of a pathology.

The final model chosen for the study was the best-performing implementation of the previously decided ResNet-18 architecture in terms of qualitative results.

### GradCAM methods

In this work, a systematic evaluation of GradCAM-based saliency methods was performed in veterinary imaging. To maximise reproducibility and avoid implementation bias, we limited our benchmark to saliency algorithms already implemented in the PyTorch-Grad-CAM repository^24^. The methods available in the PyTorch-Grad-CAM are well-tested, open-source implementations utilized by numerous XAI researchers. At the time of this study named library contained exactly eleven GradCAM-derived methods (GradCAM^23,24^, HiResCAM^24,25^, GradCAMElementWise^24^, GradCAM++^24,26^, XGradCAM^24,27^, AblationCAM^24,28^, ScoreCAM^24,29^, EigenCAM^24,30^, EigenGradCAM^24^, LayerCAM^24,31^, FullGrad^24,32^). When differentiating between CAM methods, it is essential to understand their underlying concept of inner workings and their respective differences. Class activation mapping (CAM) is a method designed to highlight regions within an image that have the most significant influence on CNN classification decision, based on spatial information maintained throughout the convolutional layers^39^. However, the original CAM method proved to be too specialized, requiring a specific CNN architecture that contains a global average pooling (GAP) layer to work. Thus, a sister method was introduced to tackle the problem of its predecessor. GradCAM, being such a method, is a generalization of CAM that does not require the presence of a GAP layer, with the authors specifying that “we can expect the last convolutional layers to have the best compromise between high-level semantics and detailed spatial information”^23,40^.

GradCAM operates by calculating a weighted average of the feature maps from the model’s final convolutional layer, based on the gradients of the target class to highlight regions that significantly influence the model’s predictions^41^. This baseline method is computationally efficient and has been widely adopted for its ability to provide intuitive visual explanations for model decisions^42,43^. However, one of its greater limitations lies in its ability to localize multiple objects or complex features within an image accurately.

Because each evaluated method is based on the operating principle of the GradCAM baseline, the proposed Table 5 indicates the necessary attributes and their respective differences.

In addition, digitization is directly related to the IT infrastructure for the successful implementation of medical digital solutions in e-health, m-health, telemedicine.

**Table 5:** Comparison of various CAM methods and their differences from GradCAM.

Method	Core concept	How it differs from GradCAM
GradCAM	Calculates a weighted average of 2D activations by the average gradient to pinpoint image regions most influential for the classification	Baseline method, computationally efficient
HiResCAM	Similar to GradCAM, but uses element-wise multiplication of activations and gradients. This may offer more detailed visualizations compared to the averaging approach in GradCAM	Aims for potentially improved localization by considering the influence of each pixel’s activation on the gradient
GradCAM ElementWise	Like GradCAM, but emphasizes positive gradients by multiplying activations and gradients followed by a ReLU operation	Addresses limitations of GradCAM in visualizing multiple pathologies by emphasizing areas with strong positive influence on the classification decision
GradCAM++	Uses second-order gradients for a more theoretically grounded approach to gradient attribution	Aims for more accurate and interpretable explanations compared to GradCAM by considering the curvature of the decision surface
XGradCAM (Axiom-based GradCAM)	Scales gradients by normalized activations	Potentially refines the gradient attribution process by considering the relative strength of activations in different regions
AblationCAM	Analyzes the importance of image regions by measuring the output drop when activations are zeroed out	Unique perspective compared to gradient-based methods. Focuses on understanding how removing specific image parts affects the model’s prediction
ScoreCAM	Uses forward activation scores to weight feature maps, avoiding gradient dependence and aiming for fairer explanations	Alternative approach less reliant on gradients. Evaluates the impact of feature maps on the model’s prediction by introducing noise based on activation values
EigenCAM	Highlights features crucial for the model’s decision by using the first principal component of activation maps, without relying on class labels	Different approach, focuses on identifying the most prominent patterns within the feature maps that contribute to the classification
EigenGradCAM	Combines GradCAM’s localization with eigenvalue decomposition (like EigenCAM but with class discrimination)	Aims to address limitations of solely gradient-based methods by incorporating information about the overall structure of the feature maps
LayerCAM	Spatially weights the activations by positive gradients. This method might be particularly effective in lower network layers	Aims for improved localization accuracy, especially in earlier layers where spatial information is more prominent
FullGrad	Considers contributions of all network neurons (including biases) to the prediction by computing and summing their gradients	Provides a comprehensive view of features influencing the model’s output, including potential biases

### Questionnaire

“An online questionnaire was devised and handed out to expert veterinarians to systematically evaluate and compare eleven CAM-based saliency methods in veterinary imaging. Three practising small-animal veterinarians served as heat-map evaluators. Evaluator 1, DVM, PhD, Professor at the University of Padua, has over 15 years of clinical radiology experience and over 50 peer-reviewed publications in veterinary imaging. Evaluator 2, DVM, PhD candidate at the University of Padua, contributes to AI-imaging research and has 5 years of thoracic-radiograph interpretation experience with over 20 publications. Evaluator 3, DVM, has 2 years of diagnostic-imaging practice and is a co-author on peer-reviewed imaging studies. None of the annotators are board-certified radiologists, yet all work daily in a tertiary referral setting where thoracic X-rays constitute a substantial portion of the caseload. Their combined publication record and routine clinical exposure meet current recommendations that saliency-map evaluations be performed by domain experts familiar with the target modality. The objective for the annotators was to assess the effectiveness of each method in accurately localizing and highlighting relevant pathological regions within canine radiographs.”

**Fig. 21:**
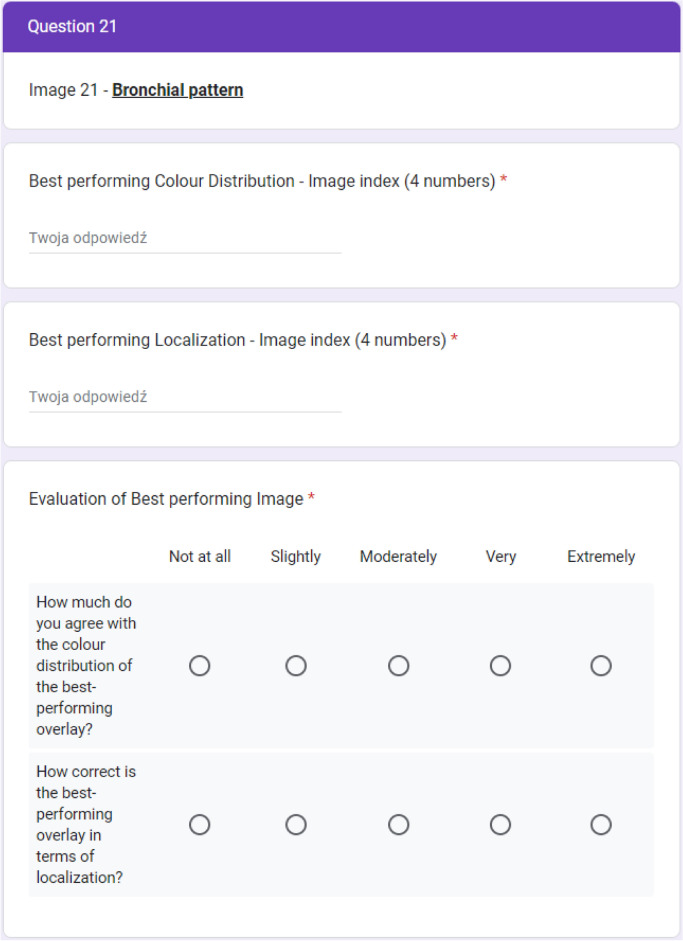
Questionnaire, choosing best performing image (Image 21 - Bronchial pattern).

**Fig. 22:**
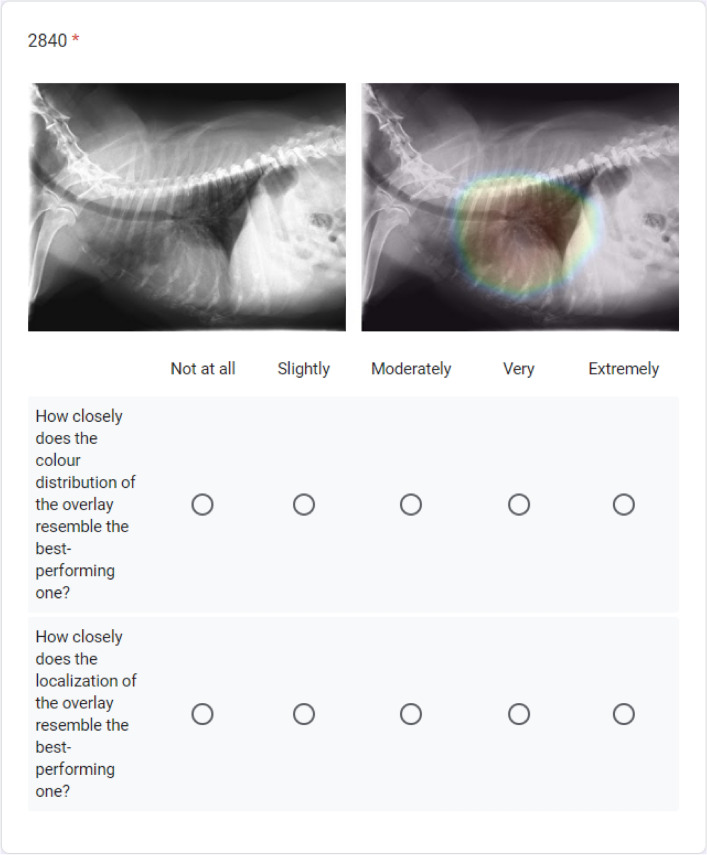
Questionnaire, relative grading criteria (Image 21 - Bronchial pattern).

Due to platform constraints, the survey, comprising of 54 sections, was separated into three parts, with each section maintaining a consistent structure but featuring different image sets. Each section was dedicated to a lesion class identified in the dataset; participants were presented with the name of the lesion at the top of the page, and eleven pairs of images were displayed in random order below. Each pair consisted of the original X-ray image on the left and the same image overlaid with a heatmap generated by one of the eleven GradCAM methods on the right. The layout and structure of the questionnaire are shown in Additional Information Section A, Figs. 21, 22.

The heatmaps were color-coded to represent the neural network’s decision-making significance, with a gradient ranging from dark red (indicating high significance) through yellow, green, and blue to transparency (indicating no significance). The participants were instructed to perform several tasks for each section:

First, they were asked to select the best-performing overlay in terms of color distribution and localization accuracy. For color distribution, they identified the overlay that best represented the significance levels of the pathological regions through its color gradation. For localization accuracy, they chose the overlay that most accurately highlighted the anatomical locations pertinent to the lesion. Participants recorded their selections by entering the image index associated with each chosen overlay. It was permissible for the same overlay to be selected for both criteria or for different overlays to be chosen for each.

Second, participants evaluated the best-performing selected overlays by rating their agreement with the color distribution and the correctness of localization. They evaluated how well the overlay’s color gradation aligned with their expert understanding of the lesion’s significance and how accurately the overlay’s highlighted regions corresponded to the anatomical areas they would examine to confirm the lesion’s presence. Ratings were provided on a 5-point Likert scale ranging from 1 (Not at all) to 5 (Extremely).

Third, participants compared each of the ten remaining overlays to the best-performing overlay based on similarity in color distribution and localization. They rated how closely each overlay’s color representation and highlighted regions matched those of the best-performing overlay, using the same 5-point Likert scale. Participants were instructed to select “Extremely” if the overlay being rated was the one they had previously identified as the best-performing in that category.

To ensure comprehensive data collection and maintain the integrity of the comparative analysis, participants were required to answer all questions before proceeding to the next section. An illustrative example was provided at the beginning of the questionnaire to familiarize participants with the format and expectations.

### Metrics: model evaluation

During the model training phase of this study, multiple metrics were used, most notably the receiver operating characteristic curve (ROC curve) as well as the confusion matrix, with their uses and importance to the study indicated below.

The receiver operating characteristic (ROC) curves, which are a graphical representation of the True Positive Rate (TPR) plotted against the False Positive Rate (FPR)^44^, while typically used for binary classification problems, can be adapted for multi-class scenarios. In this study, a one-vs-rest strategy was employed, where each of the 10 classes was independently evaluated against all other combined classes, which led to the generation of an ROC curve for each class. The area under the curve (AUC) of each ROC curve served as a performance metric, summarizing the model’s ability to discriminate/differentiate between the target class and the remaining classes. Plotting at various classification thresholds allowed for fine-tuning of class-specific threshold levels, with higher AUC values indicating better classification ability^45^.

The confusion matrix, also known as the error matrix, is a term used to represent a two-by-two-sized matrix that displays the number of true positives (TP), true negatives (TF), false positives (FP), and false negatives (FN) in each box^46^. Its evaluation during model training is crucial for establishing the accuracy of a classification model, as it can indicate the over- (FPs) or under- (FNs) sensitivity of a model.

### Metrics: CAMs’ evaluation

A series of statistical tests were conducted to evaluate the effectiveness of CAM methods correctly. These included analysis of variance (ANOVA), pair-wise t-tests whose p-values were adjusted with the Benjamini–Hochberg false-discovery-rate (FDR) procedure, Kolmogorov-Smirnov (KS) test, and Kendall’s rank correlation coefficient. Together, these tests allowed for a deeper understanding of the differences in explainability scores, consistency of results, and preferences regarding CAM methods.

ANOVA was first used to determine whether there were statistically significant differences in mean scores between CAM methods for each pathology class. By comparing the variability in scores between groups (CAM methods) to the variability within groups (annotator evaluations), this test revealed whether any method significantly outperformed the others. To identify classes in which differences between methods were statistically significant, a* p*-value threshold of 0.05 was used. The test results served as a precursor towards more detailed analyses of classes that satisfied the* p*-value threshold criteria.

For pathology classes in which the ANOVA test indicated a global difference, pair-wise t-tests were performed to identify the specific CAM methods that showed significant differences in performance. Each test compared the mean explainability scores of two methods, thereby revealing their relative strengths and weaknesses. Because every class involved $$\left( {\begin{array}{c}11\\ 2\end{array}}\right) =55$$ comparisons, the resulting p-values were adjusted with the Benjamini–Hochberg FDR procedure at $q = 0.05$. This correction was selected after exploratory trials with Bonferroni and Holm adjustments: while all three approaches controlled Type I error, Bonferroni and Holm proved overly conservative and markedly reduced statistical power, whereas FDR maintained a more favourable balance between false-positive control and sensitivity.

The Kolmogorov-Smirnov test was used to test the consistency of explainability in the eyes of clinicians of the best-performing methods. This non-parametric test compared the distributions of scores between methods in order to determine if some produced more consistent outputs. Methods with lower* p*-values in this test were identified as having significantly different outcome distributions, putting into question their variability and reliability.

Finally, the Kendall rank correlation coefficient was calculated to measure the agreement between the annotators. Unlike parametric correlation methods, Kendall’s Tau considers the concordance or discordance of paired rankings, thus quantifying how consistently annotators rated CAM methods. This provides a measure of inter-rater reliability, with higher scores indicating a more substantial agreement and reflecting shared perceptions among annotators rather than individual biases.

### Animal ethics approval

All experiments were performed in accordance with relevant guidelines and regulations. This retrospective study, which utilized data derived from routine clinical activity, was conducted in compliance with Italian law 26/2014 (transposing EU directive 2010/63/EU). Due to the retrospective nature of the study, ethical approval, related to the analysis of the data, was waived by the University of Padua, Department of Animal Medicine, Production and Health. Informed consent regarding personal data processing was obtained from all patients’ owners.

### Human ethics approval

This study also involved data collected from human participants through a questionnaire. All procedures were conducted in accordance with relevant guidelines and regulations. The study protocol was reviewed and approved by the Bioethics Committee of the University of Padua, Department of Animal Medicine, Production and Health. Prior to participation, all respondents were fully informed about the nature and purpose of the study and provided their informed consent, either written or verbal, for the use of their responses in this study (Tables 6, 7 and 8).

**Table 6:** Significant pairwise t-test FDR-adjusted* p*-values (p_adj < 0.05) for methods in the Cardiomegaly class.

Method 1	Method 2	p_adj
XGradCAM	EigenCAM	0.0022
XGradCAM	EigenGradCAM	0.00036
GradCAM	EigenCAM	0.008
GradCAM	EigenGradCAM	0.0015
EigenCAM	FullGrad	0.000006
EigenCAM	LayerCAM	0.047
EigenCAM	AblationCAM	0.043
FullGrad	LayerCAM	0.008
FullGrad	GradCAM++	0.0043
FullGrad	ScoreCAM	0.019
FullGrad	AblationCAM	0.013
FullGrad	EigenGradCAM	0.000001
FullGrad	GradCAM-ElementWise	0.011
FullGrad	HiResCAM	0.004
LayerCAM	EigenGradCAM	0.011
GradCAM++	EigenGradCAM	0.037
ScoreCAM	EigenGradCAM	0.013
AblationCAM	EigenGradCAM	0.011
EigenGradCAM	GradCAM-ElementWise	0.03

**Table 7:** Significant pairwise t-test FDR-adjusted* p*-values (p_adj < 0.05) for methods in the Alveolar Pattern class.

Method 1	Method 2	p_adj
AblationCAM	XGradCAM	0.019
AblationCAM	ScoreCAM	0.0033
AblationCAM	EigenCAM	0.0068
EigenGradCAM	FullGrad	0.013
EigenGradCAM	XGradCAM	0.000002
EigenGradCAM	GradCAM	0.011
EigenGradCAM	HiResCAM	0.00065
FullGrad	ScoreCAM	0.00026
FullGrad	EigenCAM	0.00046
XGradCAM	LayerCAM	0.00011
XGradCAM	GradCAM++	0.00065
XGradCAM	ScoreCAM	0.000000
XGradCAM	EigenCAM	0.000000
XGradCAM	GradCAM	0.036
XGradCAM	GradCAM-ElementWise	0.031
LayerCAM	HiResCAM	0.011
GradCAM++	ScoreCAM	0.012
GradCAM++	EigenCAM	0.024
ScoreCAM	GradCAM	0.00011
ScoreCAM	HiResCAM	0.000005
ScoreCAM	GradCAM-ElementWise	0.0064
EigenCAM	GradCAM	0.00022
EigenCAM	HiResCAM	0.000006
EigenCAM	GradCAM-ElementWise	0.011

**Table 8:** Significant pairwise t-test FDR-adjusted p-values (p_adj < 0.05) for methods in the Bronchial Pattern class.

Method 1	Method 2	p_adj
GradCAM	EigenCAM	0.047
EigenCAM	AblationCAM	0.048
EigenCAM	XGradCAM	0.000006
EigenCAM	FullGrad	0.00027
EigenCAM	GradCAM-ElementWise	0.015
XGradCAM	GradCAM++	0.00065
XGradCAM	LayerCAM	0.00011
XGradCAM	ScoreCAM	0.00011
XGradCAM	EigenGradCAM	0.000006
GradCAM++	FullGrad	0.011
LayerCAM	FullGrad	0.0024
FullGrad	ScoreCAM	0.0021
FullGrad	EigenGradCAM	0.00029
GradCAM-ElementWise	EigenGradCAM	0.017

## Data Availability

The data sets generated during and/or analysed during the current study are not publicly available because they are the property of the Veterinary Teaching Hospital of the University of Padua but are available from the corresponding author upon reasonable request.

## C - Python code repository

The entirety of the code used for this article, including data mining, model training, model testing, heatmap overlaying, and statistical analyses of the questionnaire, can be found in this GitHub repository.

https://github.com/pddusza/CAM-analysis
